# Differentiation between wild boar and domestic pig in food by targeting two gene loci by real-time PCR

**DOI:** 10.1038/s41598-019-45564-7

**Published:** 2019-06-25

**Authors:** Maria Kaltenbrunner, Walter Mayer, Kirsten Kerkhoff, Rita Epp, Hermann Rüggeberg, Rupert Hochegger, Margit Cichna-Markl

**Affiliations:** 1 Austrian Agency for Health and Food Safety, Institute for Food Safety Vienna, Department of Molecular Biology and Microbiology, Spargelfeldstraße 191, 1220 Vienna, Austria; 2 Department of Analytical Chemistry, Faculty of Chemistry, University of Vienna, Währinger Straße 38, 1090 Vienna, Austria; 3 Impetus GmbH & Co. Bioscience KG, Fischkai 1, 27572 Bremerhaven, Germany

**Keywords:** Molecular biology, Genetics

## Abstract

Studies indicate that many meat products are not authentic, most frequently because the meat species differ from those given on the food labels. At present, DNA based methods play the most important role in meat species authentication. Discrimination of wild boar and domestic pig meat in food is challenging because it is differentiation on the subspecies level. We developed and validated two singleplex real-time PCR assays targeting SNP rs81416363 on chromosome 9 and a duplex real-time PCR assay targeting SNP g.299084751 C > T in the *NR6A1* gene located on chromosome 1. The singleplex real-time PCR assays led to some ambiguous results for Mangalica and Krškopolje pig breeds and wild boar individuals from Germany, the duplex real-time PCR assay particularly for the Turopolje pig breed. We demonstrate that the probability of misclassification can be substantially reduced if the results of both the singleplex real-time PCR assays and the duplex real-time PCR assay are taken into consideration. 86 (91.5%) of a total of 94 individuals, comprising 64 domestic pigs (14 different breeds and 6 cross-breeds) and 30 wild boars (from Austria, Germany, Romania, USA and Estonia), were classified correctly.

## Introduction

Food adulteration has become a global issue. The term “food adulteration” refers to any kind of deliberately false or misleading statement on the food label, regarding e.g. quality, composition, geographic origin or type of food processing. Recently, Stamatis *et al*. analysed 384 commercial food products of animal origin, including milk, cheese, processed meat and frozen fish products, to investigate whether the declaration of the animal species corresponded with the actual composition of the product. 16 out of 86 processed meat products were found to be adulterated by removal, 7 by addition and 5 by substitution of species^1^. This finding is in concordance with previous papers hinting at meat product adulteration with respect to the species the meat originates from^2–4^.

Over the last centuries, pig has been the most frequently consumed animal species in Western societies. Since meat from game animals is leaner and therefore healthier than meat from domesticated animal species, there is an increasing trend to market meat from European wild boar (*Sus scrofa scrofa*) in addition to meat from domestic pig (*Sus scrofa domesticus*). Advertised as delicacy, steaks, bacon, sausages and salami from wild boar are more expensive than the respective products from domestic pig. Thus, some meat producers could try to increase their profit by replacing (part of) wild boar meat by pork. However, nowadays adulteration the other way round - substitution of pork by wild boar meat - could also happen. This is due to outbreaks of African swine fever in North-Eastern Europe, in particular Lithuania, Latvia, Estonia and Poland, in 2014 and 2015^5^. In order to reduce the chance of spreading this highly contagious epidemic, numerous wild boars have been slaughtered in these regions of Europe and in neighbouring countries. Due to a sharp decline in price, wild boar meat is currently difficult to market and thus meat producers could be tempted to intentionally replace pork by wild boar meat.

Numerous DNA based methods already exist for the detection of meat from domestic pig in commercial food products. These methods can be applied e.g. to verify if food products declared to be “halal” actually do not contain pork. Discrimination of wild boar and domestic pig meat in food is, however, more challenging, because it is differentiation on the subspecies level. The genomes of wild boar and domestic pig are highly homologous and thus, the number of subspecies-specific bases is very low. Hybridisation and back-crossings, occurred either intentionally (during the domestication process) or accidentally (by outdoor farming of domestic pigs), resulted in even higher sequence homologies and intra-subspecies variability^6,7^.

DNA based methods published so far target polymorphisms in genes that have been selected in the process of wild boar domestication and are associated with traits like coat colour, body composition, reproduction and behaviour^8^, e.g. *melanocortin 1 receptor* (*MC1R*), *v-kit Hardy-Zuckerman 4 feline sarcoma viral oncogene homolog* (*KIT*), *insulin like growth factor-2* (*IGF2*), *ryanodine receptor 1* (*RYR1*) and *nuclear receptor subfamily 6 group A member 1* (*NR6A1*). Most frequently, analysis is based on polymerase chain reaction-restriction fragment length polymorphism (PCR-RFLP). The applicability of PCR-RFLP in routine food analysis is, however, limited because it is rather laborious and time consuming and not suitable for the analysis of complex food products. The only real-time PCR assay published so far is a duplex assay targeting a single nucleotide polymorphism (SNP) in *MC1R*. The duplex real-time PCR assay involved one forward primer and one reverse primer for amplification of the target region in both wild boar and domestic pig. For differentiation, two TaqMan probes were used, being specific for the alleles in wild boar and domestic pig, respectively^9^. Wild boar samples could be identified correctly, but for the domestic pig breed Duroc and cross-breeds thereof, the results were ambiguous.

In the present study, we developed and validated two singleplex real-time PCR assays targeting SNP rs81416363 on chromosome 9 and a duplex real-time PCR assay targeting SNP g.299084751 C > T in the *NR6A1* gene on chromosome 1. We demonstrate that the probability of misclassification can be substantially reduced if the results of both the singleplex real-time PCR assays and the duplex real-time PCR assay are taken into consideration.

## Results

### Primer and probe design

**Figure 1:**
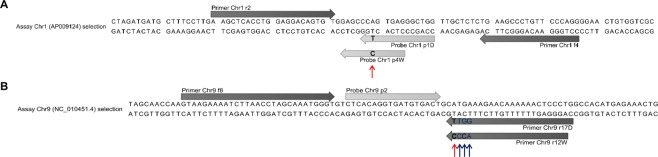
Partial sequences of (**A**) the *NR6A1* gene on chromosome 1 carrying SNP g.299084751 C > T, targeted by the duplex assay “assay_Chr1_”, and (**B**) chromosome 9 carrying the SNP g.118314929 A > G (rs81416363), targeted by the two singleplex assays “assay_Chr9*D*_” and “assay_Chr9*W*_”. Arrows indicate the positions of the primers (dark gray) and probes (light gray). (**A**) Duplex assay_Chr1_ included forward primer Chr1f4, reverse primer Chr1r2, probe Chr1p4_*W*_ and probe Chr1p1_*D*_. (**B**) Singleplex assay_Chr9*D*_ included forward primer Chr9f8, reverse primer Chr9r17_*D*_ and probe Chr9p2; singleplex assay_Chr9*W*_ included forward primer Chr9f8, reverse primer Chr9r12_*W*_ and probe Chr9p2. Subspecies-specific bases are shown in bold (vertical red arrows) and mismatch bases in blue (vertical blue arrows).

For primer and probe design, we selected polymorphisms that had already been reported to allow the discrimination of wild boar and domestic pig. We started with the SNP g.299084751 C > T in the *NR6A1* gene, which has been found to be associated with the number of thoracic and lumbar vertebrae: wild boars, which are homozygous for the wild type allele g.299084751 C, have 19 vertebrae, whereas domestic pigs, which are homozygous for g.299084751 T, have 21–23 vertebrae. In the study of Fontanesi *et al*., this SNP was used to discriminate wild boar and domestic pig by PCR-RFLP^10^. We aimed to design one primer pair for amplification of the target region in both wild boar and domestic pig, and two TaqMan probes, specific for the wild boar allele (g.299084751 C) and the domestic pig allele (g.299084751 T), respectively. The duplex assay should make it possible to detect wild boar and domestic pig simultaneously in one and the same well. In total, we designed four forward primers, three reverse primers, four wild boar probes and one domestic pig probe and combined them to 21 primer/probe systems (Chr1a - u, Supplementary Table 1). Primer/probe system Chr1a targeted the upper strand, primer/probe systems Chr1b-u the lower strand of the *NR6A1* gene. In a series of experiments, we investigated if the primer/probe systems were specific for wild boar or showed cross-reactivity with domestic pig breeds/cross-breeds. Primer/probe system Chr1a resulted in a ΔCt value ≥ 10.56 between domestic pig and wild boar (Supplementary Table 2). For primer/probe systems Chr1i – o, ΔCt values ranged from 8.65 to 11.19. Primer/probe systems Chr1b – h and Chr1p – t did not result in an increase in the fluorescence signal for domestic pig breeds/cross-breeds. Since primer/probe system Chr1q resulted in the lowest Ct value (24.77; n = 2) for wild boar, we tested its applicability in combination with a TaqMan probe for domestic pig in the duplex assay format (primer/probe system Chr1u, Supplementary Table 1). Since the Ct values for both wild boar (26.21, n = 2) and domestic pig (27.90, n = 2) were satisfactory, all further PCR experiments targeting SNP g.299084751 C > T were performed with primer/probe system Chr1u. In the following, the duplex real-time PCR assay is called assay_Chr1_, consisting of the assay for domestic pig, assay_Chr1*D*_, involving forward primer Chr1f4, reverse primer Chr1r2 and probe Chr1p1_*D*_, and the assay for wild boar, assay_Chr1*W*_, involving forward primer Chr1f4, reverse primer Chr1r2 and probe Chr1p4_*W*_ (Fig. 1A).

Since we had learned from literature that the discrimination power by targeting only one polymorphism is most probably not sufficient, we looked for a further suitable gene locus. By investigating 20 SNPs for their applicability to differentiate between wild boar and domestic pig, Beugin *et al*. had found the SNPs rs80864596 (intergenic, chromosome 5), rs80796712 (*glycogen synthase kinase 3-beta*, chromosome 13) and rs81416363 (intergenic, chromosome 9) to show the highest discrimination power^11^. We therefore selected these three SNPs for designing primer/probe systems. Compared to primer/probe design for SNP g.299084751 C > T, we pursued a different strategy. Instead of locating the subspecies-specific base in the probe, we located it at the penultimate site from the 5′ end of the forward or the reverse primer. In order to enhance the specificity, we introduced deliberate base mismatches. This so-called mismatch amplification mutation assay (MAMA) technique^12,13^ has the advantage that it is rather cost efficient because one and the same probe can be tested with a number of primers differing in the mismatch position. In the first series of experiments we introduced the mismatch base either at the 3^rd^ or the 6^th^ position from the 3′ end of the primer carrying the subspecies specific base. When we had developed real-time PCR assays for red deer, fallow deer and sika deer, respectively^14–16^, this strategy had turned out to be successful. However, in the present study none of the primer/probe systems (Chr5b – d, Chr13b – c, Chr9b – g, Chr9i – n, Supplementary Table 1) allowed the differentiation between domestic pig and wild boar (Supplementary Table 3). The best results (ΔCt value between domestic pig breeds/cross-breeds and wild boar ≥5.24) were obtained with primer/probe system Chr9j. In this primer/probe system, targeting the SNP rs81416363 on chromosome 9, the mismatch base was located in the 3^rd^ position from the 3′ end of the primer (TTCA**C**G → TTCC**C**G). In order to increase the specificity, we introduced a further mismatch in the 4^th^ (TTCA**C**G → TTGC**C**G, TTAC**C**G, TTTC**C**G) or 5^th^ position (TTCA**C**G → TACC**C**G, TCCC**C**G, TGCC**C**G) from the 3′ end of the primer. The introduction of additional mismatches turned out to be successful. With primer/probe system Chr9r (Supplementary Table 1) we obtained both the lowest Ct value for wild boar (22.09; n = 2) and the highest ΔCt value (10.52) between domestic pig breeds/cross-breeds and wild boar (Supplementary Table 3). This real-time PCR assay for wild boar, targeting the SNP rs81416363 located on chromosome 9, was based on primer/probe system Chr9r (Chr9f8, Chr9r12_*W*_, Chr9p2).

Next, we applied the MAMA strategy to develop the corresponding real-time PCR assay, targeting the SNP rs81416363 located on chromosome 9, for domestic pig. We located the domestic pig-specific base at the penultimate base from the 5′ end of the primer and the mismatch base at the 3^rd^ position from the 3′ end (primer/probe systems Chr9v – x; Supplementary Table 1). However, the introduction of one mismatch base was not sufficient to allow the differentiation of domestic pig and wild boar. The introduction of mismatch bases at the 3^rd^ and 5^th^ position from the 3′ end of the primer (primer/probe systems Chr9y - ag) led to a ΔCt value ≥ 4.83. When the mismatches were at positions 3 and 4 (primer/probe systems Chr9ah - aj), the ΔCt value was ≥5.85. By introducing three consecutive mismatch bases next to the subspecies-specific base (primer/probe systems Chr9ak - am), the specificity could be increased. Primer/probe system Chr9ak, in which the mismatches were at position 3, 4 and 5 (TTCA**T**G → TGGT**T**G) led to the highest ΔCt value of ≥9.97. Thus, all further experiments were carried out with primer/probe system Chr9ak. In the following, the two singleplex assays targeting the SNP rs81416363 on chromosome 9 are referred to as assay_Chr9*D*_, including forward primer Chr9f8, reverse primer Chr9r17_*D*_ and probe Chr9p2, and assay_Chr9*W*_, including forward primer Chr9f8, reverse primer Chr9r12_*W*_ and probe Chr9p2 (Fig. 1B).

### Optimisation of primer/probe concentration and ratio

All results presented above were obtained with primer and probe concentrations of 500 nM and 200 nM (primer/probe systems targeting chromosome 1), and 200 nM and 100 nM (primer/probe systems targeting chromosomes 5, 9 and 13), respectively. Next, we investigated, whether the specificity of the real-time PCR assays could be increased by optimising the primer/probe concentration and ratio. In case of assay_Chr1_, the optimal primer and probe concentrations were found to be 1,000 nM and 200 nM, respectively (Supplementary Table 4). In case of assay_Chr9*W*_ and assay_Chr9*D*_, a surplus of the primer carrying both the subspecies-specific base and the mismatch bases resulted in higher ΔCt values between the cross-reacting subspecies and the target species. The highest selectivity of assay_Chr9*W*_ and assay_Chr9*D*_ was achieved with forward primer/reverse primer/probe concentrations of 12.5/200/50 nM and 62.5/800/50 nM, respectively.

### Cross-reactivity tests

**Figure 2:**
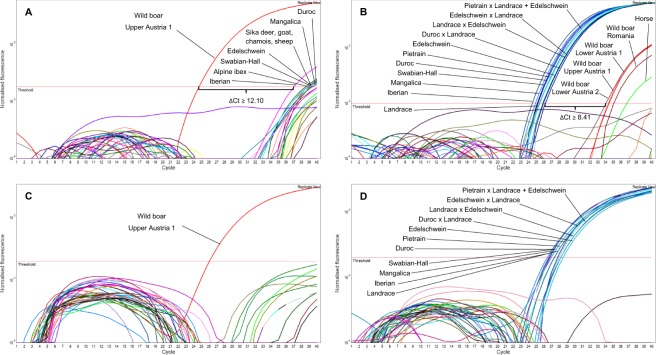
Results obtained by performing cross-reactivity tests with DNA isolates (10 ng/µL) from 22 animal species and wild boar/domestic pig. (**A**) assay_Chr9*W*_, (**B**) assay_Chr9*D*_, (**C**) assay_Chr1*W*_ and (**D**) assay_Chr1*D*_.

Next, we performed cross-reactivity tests with DNA isolates from wild boar, various domestic pig breeds/cross-breeds and further 22 animal species listed in Supplementary Table 5. Figure 2A–D show the amplification curves obtained with assay_Chr9*W*_, assay_Chr9*D*_, assay_Chr1*W*_ and assay_Chr1*D*_, respectively. Assay_Chr9*W*_ showed slight cros*s*-reactivity with domestic pigs, sika deer, alpine ibex, sheep, goat and chamois (ΔCt ≥ 12.10, n = 2), assay_Chr9*D*_ with wild boars, sika deer, moose, horse and turkey (ΔCt ≥ 8.41, n = 4). In contrast to the assays targeting the SNP rs81416363 on chromosome 9, assay_Chr1_ did not show any cross-reactivity (with the exception of assay_Chr1*W*_ for roe deer in only one out of four replicates). We also analysed DNA isolates from 50 plant species commonly used as food ingredients (Supplementary Table 6). None of the assays was found to show cross-reactivity with any of the plant species.

### Screening various commercial master mixes for their applicability

In a next series of experiments, we investigated whether the type of the commercial master mix influenced the selectivity of the assays. We analysed DNA isolates from 23 animal species including 3 wild boar individuals and 11 breeds/cross-breeds of domestic pig with a total of five master mixes from different suppliers. The master mixes and the respective temperature programs are given in Supplementary Table 7. In general, the type of the master mix influenced both the absolute Ct values and the ΔCt values between target and cross-reacting (sub)species. However, the real-time PCR assays were influenced to a different extent. In case of assay_Chr9*W*_, for example, mix 2 and 3 resulted in higher Ct values than master mix 1 (the standard master mix in the present study), 4 and 5 (Ct ± SD (n = 6): mix 1: 25.03 ± 0.15; mix 2: 31.17 ± 1.70; mix 3: 28.43 ± 0.13; mix 4: 26.64 ± 0.10; mix 5: 26.64 ± 0.17). In case of assay_Chr9*D*_, the type of master mix had an influence on the ΔCt values and thus the selectivity of the assay. ΔCt values obtained with master mix 1, 2, 3 and 4 were ≥ 8.41, ≥ 10.40, ≥ 7.18 and ≥ 5.86, respectively, whereas with master mix 5, no cross-reactivity was observed at all. In case of assay_Chr1_, rather similar Ct values were obtained with master mix 1, 3 and 4. However, master mix 2 and 5 did not result in the formation of any products, most probably because these mixes are not suitable for multiplexing. Based on these findings, we highly recommend to test the applicability if another type of master mix is intended to be used.

### Robustness

The robustness of the real-time PCRs assays was investigated by varying the annealing temperature ±1 °C and the volume of the reaction mix per well by ±5% and by applying two different real-time PCR cyclers. The experiments were performed with DNA isolates from wild boar, domestic pig and roe deer. For assay_Chr9*W*_ and assay_Chr9*D*_, the Ct values were very similar to those obtained under standard conditions (ΔCt values <1.00) except when the annealing temperature was increased to 61 °C (ΔCt values ≤2.16 and ≤1.57, respectively). With ΔCt values ≤0.77, assay_Chr1_ turned out to be even more robust. Neither down-scaling the total reaction volume nor the use of another real-time PCR cycler substantially influenced the Ct values, demonstrating the robustness of the real-time PCR assays.

### Working range, linear range and amplification efficiency

**Figure 3:**
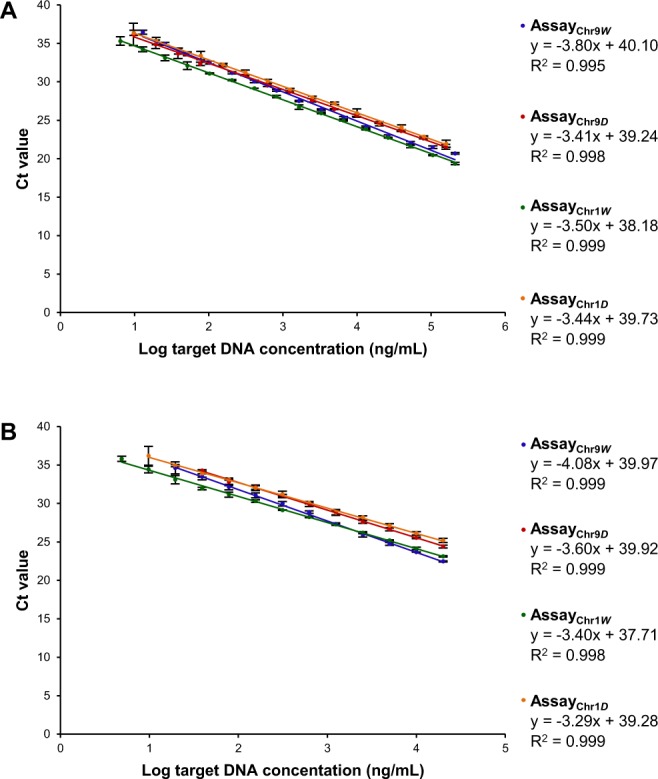
Range of linearity of the four assays, (**A**) determined in water as background and (**B**) determined in herring sperm DNA as non-target background DNA.

By analysing a wild boar DNA isolate (211 µg/mL) and two domestic pig DNA isolates (158 µg/mL and 160 µg/mL) serially diluted in water (1:2–1:524,288), assay_Chr9*W*_, assay_Chr9*D*_, assay_Chr1*W*_ and assay_Chr1*D*_ were linear between 211 µg/mL and 13 ng/mL (R^2^ = 0.9948), 158 µg/mL and 10 ng/mL (R^2^ = 0.9981), 211 µg/mL and 6 ng/mL (R^2^ = 0.9994) and 160 µg/mL and 10 ng/mL (R^2^ = 0.9988), respectively (Fig. 3A). The amplification efficiencies calculated from the slopes of the standard curves were 83%, 96%, 93% and 95%, respectively. In addition, the range of linearity was evaluated by analysing wild boar and domestic pig DNA isolates serially diluted in herring sperm DNA. For assay_Chr9*W*_ and assay_Chr9*D*_, linearity ranged from 20 µg/mL to 20 ng/mL (R^2^ = 0.9988) and 20 µg/mL to 39 ng/mL (R^2^ = 0.9985), respectively (Fig. 3B). In case of assay_Chr1*W*_ and assay_Chr1*D*_, linearity was in the range from 20 µg/mL to 5 ng/mL (R^2^ = 0.9978) and 20 µg/mL to 10 ng/mL (R^2^ = 0.9988), respectively. The amplification efficiencies calculated from the slopes of the standard curves were 76%, 90%, 97% and 101%, respectively.

These results indicate that the working range of the real-time PCR assays is almost identical, whereas the range of linearity is narrower for the assays targeting chromosome 9 than for those targeting chromosome 1. All amplification efficiencies were between 90% and 110%, as recommended by the ENGL guidelines^17^, except for assay_Chr9*W*_ (83% in water, 76% in herring sperm DNA). The lower amplification efficiency of assay_Chr9*W*_ is most probably caused by decreased primer binding stability due to the mismatches, introduced to increase the selectivity for the target animal species.

### Limit of detection (LOD) in herring sperm DNA and pig DNA as background DNA

The LOD was defined as the lowest DNA concentration that resulted in an increase in the fluorescence signal in at least 19 out of 20 replicates. In addition, the Ct value should be below the Ct value obtained for cross-reacting species.

**Figure 4:**
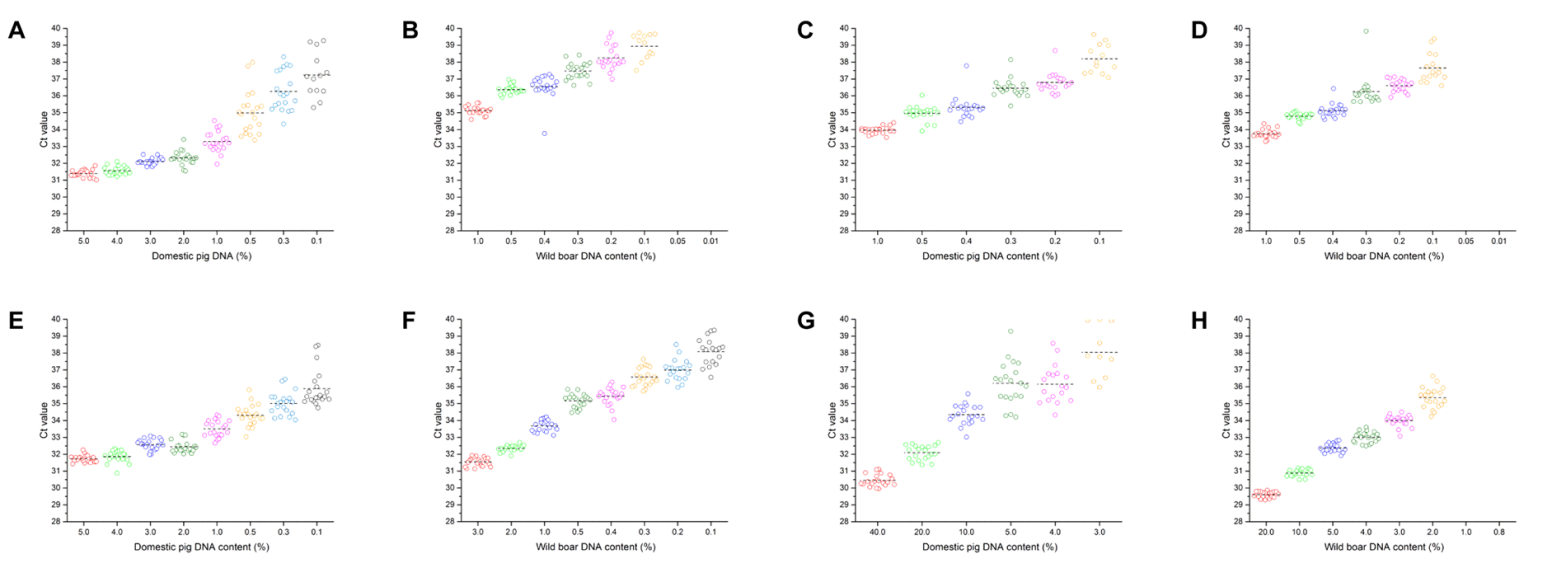
Determination of the LOD by analysing serial dilutions of (**A**–**D**) DNA mixtures containing wild boar or domestic pig DNA in herring sperm DNA and (**E**–**H**) a DNA mixture containing wild boar DNA in domestic pig DNA or domestic pig DNA in wild boar DNA as background DNA. (**A**,**E**) were obtained by using assay_Chr9*D*_, (**B**,**F**) by using assay_Chr9*W*_, (**C**,**G**) by using assay_Chr1*D*_ and (**D**,**H**) by using assay_Chr1*W*_. Measurements were carried out in 20 replicates. Circles represent individual Ct values, horizontal lines indicate the mean values.

In herring sperm DNA, the LOD of assay_Chr9*D*_ (1.0%, w/w, Fig. 4A) was slightly higher than the LOD of the other three assays (0.2%, w/w, Fig. 4B–D). The higher LOD of assay_Chr9*D*_ is caused by the cross-reactivity with wild boar (ΔCt value between wild boar and domestic pig only ≥8.41).

In addition, we determined the LOD of the real-time PCR assays for wild boar, assay_Chr1*W*_ and assay_Chr9*W*_, in domestic pig DNA as background, and that of the assays for domestic pig, assay_Chr1*D*_ and assay_Chr9*D*_, in wild boar DNA as background. With 2%, 0.2%, 5% and 2% (w/w), the LODs of assay_Chr9*D*_ (Fig. 4E), assay_Chr9*W*_ (Fig. 4F), assay_Chr1*D*_ (Fig. 4G) and assay_Chr1*W*_ (Fig. 4H) were rather different. The higher LOD of assay_Chr1*D*_ in the presence of wild boar DNA was caused by signal suppression, most probably due to competition of the two probes for the target sequence in the duplex assay format. To investigate signal suppression in more detail, DNA mixtures containing either 25% (w/w) wild boar DNA in domestic pig DNA or 25% (w/w) domestic pig DNA in wild boar DNA were analysed, in addition to positive controls (wild boar DNA and domestic pig DNA, respectively).

**Figure 5:**
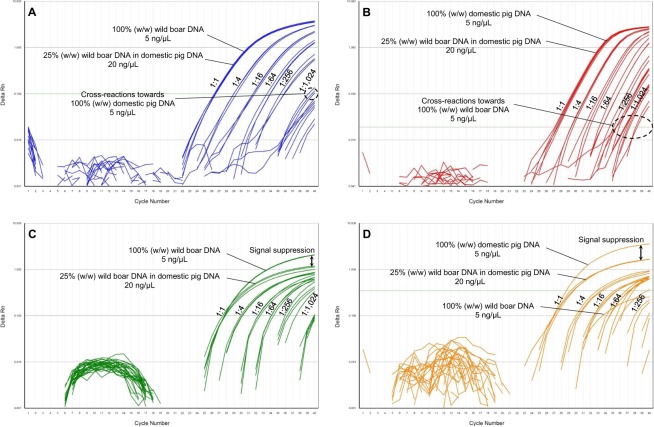
Amplification curves (in semi log view) obtained for DNA mixtures (20 ng/µL) containing 25% (w/w) of the target subspecies (wild boar or domestic pig) and 75% (w/w) of the non-target subspecies (wild boar or domestic pig) and serial dilutions thereof (1:4 to 1:1,024). DNA isolates (5 ng/µL) from wild boar and domestic pig, respectively, were used as controls. Assay_Chr9*W*_ (**A**) and assay_Chr9*D*_ (**B**) show cross-reactivity with the non-target subspecies. Assay_Chr1*W*_ (**C**) and assay_Chr1*D*_ (**D**) show signal suppression in presence of non-target subspecies.

When we compared the positive controls with the 1:4 diluted standards containing non-target DNA, we did not find a difference in the amplification curves (ΔRn values) for assay_Chr9*W*_ and assay_Chr9*D*_ (Fig. 5A,B). However, in case of assay_Chr1*W*_ and assay_Chr1*D*_, the fluorescence signals (ΔRn values) obtained for the positive control were higher than those obtained for the 1:4 diluted standard at the same target DNA concentration (Fig. 5C,D).

### Repeatability

The repeatability of the real-time PCR assays was investigated by analysing meat extract mixtures containing 30% (w/w) target DNA and 70% (w/w) non-target subspecies DNA as well as serial dilutions thereof, in duplicates five times on four different days. The RSD of the Ct values was ≤1.0% (assay_Chr1*W*_), ≤1.9% (assay_Chr9*W*_), ≤2.3% (assay_Chr1*D*_) and ≤3.5% (assay_Chr9*D*_), demonstrating the high repeatability of the assays.

### Analysis of samples from wild boar and domestic pig individuals

**Figure 6:**
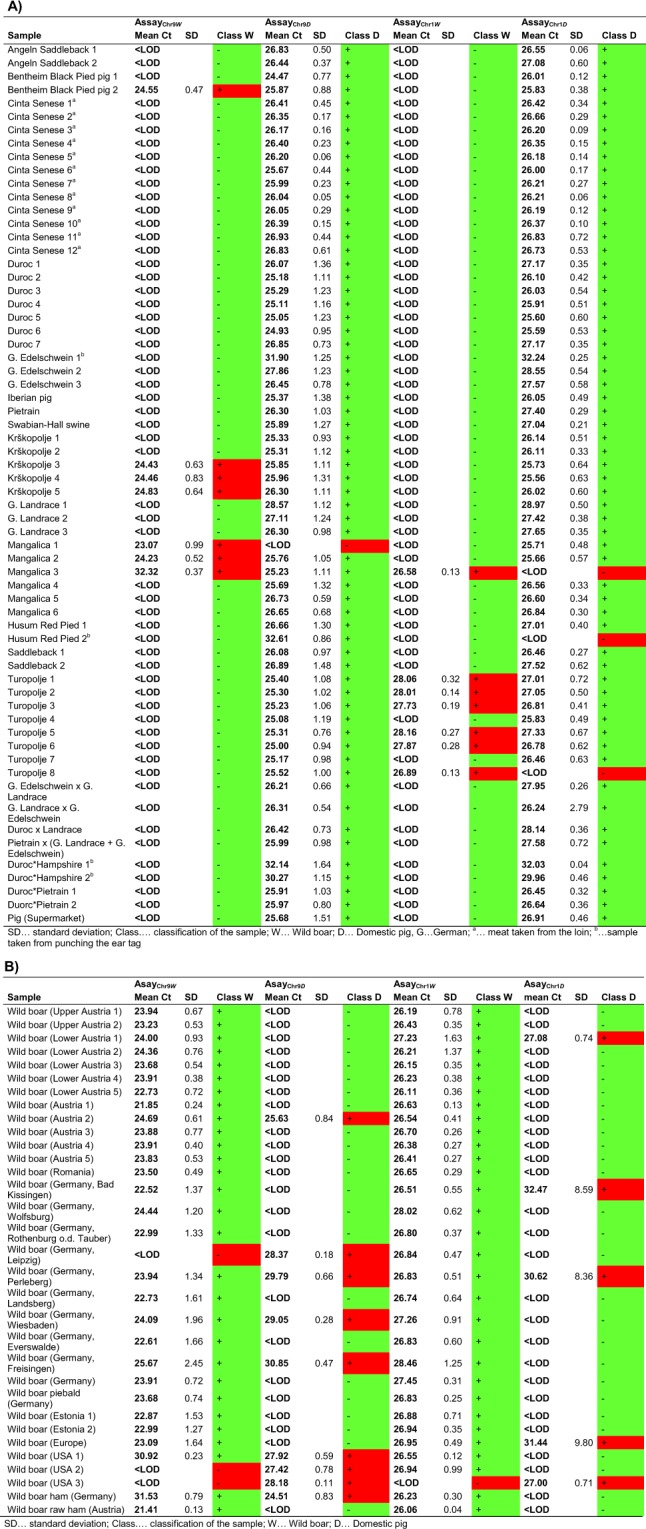
Results obtained for (**A**) 64 domestic pig and (**B**) 30 wild boar samples. Measurements were carried out in at least four replicates.

The real-time PCR assays were applied to the analysis of samples from 64 domestic pig individuals, including 14 breeds and 6 cross-breeds (Fig. 6A). In addition, we analysed a total of 30 wild boar samples from 5 different countries (Austria, Estonia, Germany, Romania and USA, Fig. 6B). In case of one wild boar individual from Europe, the exact origin was unknown. Each sample was analysed in at least four replicates. To each PCR plate, a positive control (DNA mixture, 10 ng/µL) was added, containing the target subspecies in the same concentration as the LOD, providing the cut-off Ct value.

When we analysed the positive controls in 14 replicates, the mean ± SD of the Ct values was 33.85 ± 0.93 (assay_Chr9*W*_), 35.19 ± 1.11 (assay_Chr9*D*_), 32.89 ± 0.28 (assay_Chr1*W*_) and 32.60 ± 0.47 (assay_Chr1*D*_). With the exception of assay_Chr9*D*_, all samples resulting in higher Ct values were considered <LOD. In case of assay_Chr9*D*_, for several wild boar samples Ct values ≥ 34.61 were obtained. In order to lower the probability of false positive results, we set the cut-off Ct value of assay_Chr9*D*_ at 34.00. For the application of assay_Chr9*W*_ and assay_Chr9*D*_ in routine analysis, we recommend to use a positive control consisting of 2% (w/w) domestic pig DNA, 0.2% (w/w) wild boar DNA and 97.8% (w/w) herring sperm DNA.

For the analysis of domestic pig samples with assay_Chr9*D*_, we obtained 63 positive and only one negative result (Mangalica 1) (Fig. 6). From the 12 Cinta Senese individuals, we analysed two different meat parts, a lean sample (from longissimus dorsi) and a subcutaneous back fat sample (from loin) (data not shown). Ct values obtained for lean samples (mean Ct ± SD of 25.24 ± 0.44, n = 48) were very similar to those for subcutaneous back fat samples (26.29 ± 0.35, n = 48). When we analysed the 30 wild boar samples with assay_Chr9*D*_, 21 did not result in an increase in the fluorescence signal, but nine individuals (one from Austria, five from Germany and three from USA) were identified as domestic pig.

With assay_Chr9*W*_, the majority of wild boar samples was assigned correctly, only for three samples (one from Germany and two from USA) we obtained negative results. However, assay_Chr9*W*_ led also to positive results for some domestic pig individuals, including one Bentheim Black Pied, three Krškopolje and three Mangalica pigs. Notably, the three Mangalica pigs from Austria were identified as domestic pig, whereas the three individuals from Germany were identified as wild boar. In case of assay_Chr9_, including assay_Chr9*W*_ and assay_Chr9*D*_, 12 out of the 94 meat samples led to ambiguous results, because an increase in the fluorescence signal was obtained with both real-time PCR assays. Furthermore, one Mangalica pig individual was identified as wild boar and three wild boar individuals as domestic pig.

In the study of Beugin *et al*.^11^ the suitability of the SNP rs81416363 had been tested on “pure” wild boars hunted in a natural park in the Vosges in France and a limited number of commercial pig breeds (Landrace, Large White, Piétrain and Duroc). Wild boars were found to carry the G allele (frequency 100%, no heterozygosity), domestic pigs the A allele (frequency 98%, heterozygosity 5%).

**Figure 7:**
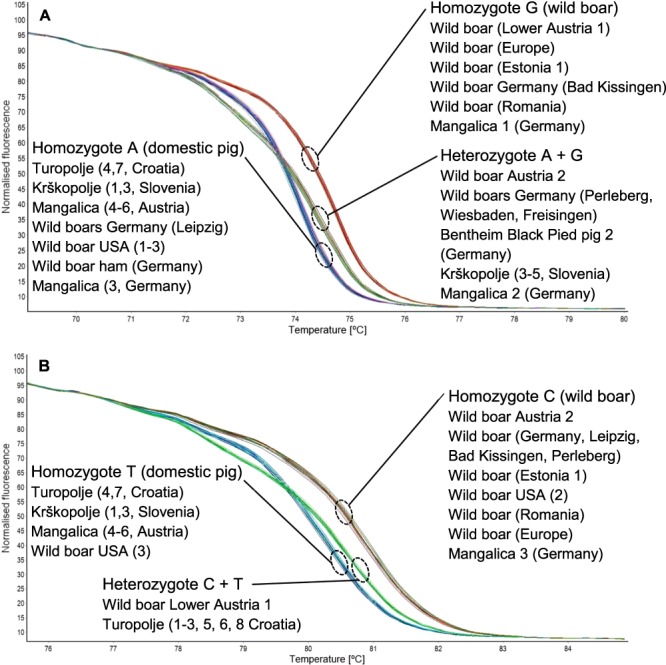
Normalised HRM curves for (**A**) SNP rs81614363 (g.118314929 A > G) on chromosome 9 for homozygote G (wild boar), homozygote A (domestic pig) and heterozygote A + G and (**B**) SNP g.299084751 C > T on chromosome 1 for homozygote C (wild boar), homozygote T (domestic pig) and heterozygote C + T.

To find out why assay_Chr9*W*_ and assay_Chr9*D*_ led to several misclassifications and some ambiguous results, we genotyped the respective wild boar and domestic pig samples by high resolution melting (HRM) analysis. We applied HRM analysis because it is a powerful, time- and cost-efficient technique for SNP genotyping^18^. The normalized melting curves (Fig. 7A) indicate that the homozygous G genotype was not only found for wild boar samples, but also for the Mangalica sample (sample 1) for which an increase in the fluorescence signal was obtained with assay_Chr9*W*_ but not with assay_Chr9*D*_. Wild boar samples resulting in an increase in the fluorescence signal with assay_Chr9*D*_ were found to carry at least one copy of the A allele. Four wild boar individuals showed the heterozygous genotype, five were homozygous for the A allele. In addition, five domestic pig individuals, including three Krškopolje individuals from Slovenia, were found to carry both the A and the G allele. Thus, by HRM genotyping we could verify that the results obtained with assay_Chr9*W*_ and assay_Chr9*D*_ were in accordance with the respective genotypes of the individuals. However, our results indicate that the two singleplex assays targeting SNP rs81416363, assay_Chr9*W*_ and assay_Chr9*D*_, do not allow the unambiguous discrimination of wild boar and domestic pig.

With assay_Chr1*D*_, all domestic pig samples except three were classified as domestic pig (Fig. 6). In accordance with assay_Chr9*D*_, Ct values obtained for lean and subcutaneous back fat samples from the 12 Cinta Senese individuals were very similar (mean Ct ± SD of 25.99 ± 0.25 (n = 48) and 26.36 ± 0.25 (n = 48), respectively). However, with assay_Chr1*D*_ also five out of the 30 wild boar samples were identified as domestic pig. The analysis of wild boar samples with assay_Chr1*W*_ led to only one negative result. However, also one Mangalica sample and six Turopolje individuals led to an increase in the fluorescence signal with assay_Chr1*W*_.

In the study of Fontanesi *et al*.^10^ the SNP g.299084751 C > T had been genotyped by analysing samples from 293 domestic pigs of five commercial breeds (Italian Large White, Italian Landrace, Italian Duroc, Belgian Landrace and Piétrain) and 201 wild boars from South Central Europe (Northern Italy) and South Eastern Europe (Slovenia and Western Balkan regions). Fontanesi *et al*. found all domestic pig individuals to be homozygous for the T allele. 7.5% of the wild boar individuals carried at least one copy of the T allele, with individuals from South East Europe more frequently (12.2%) than individuals from South Central Europe (3.6%). 11.1% of the wild boars from South East Europe were of the heterozygous C/T genotype.

When we genotyped the wild boar and domestic pig individuals for SNP g.299084751 C > T by HRM analysis, we also detected the T allele not only in domestic pigs but also in some wild boars (Fig. 7B). The wild boar individual from USA showed the homozygous T genotype whereas the wild boar individual from Lower Austria carried both the C and the T allele. The majority of the wild boar individuals was, however, homozygous for the C allele. This result indicates that the increase in the fluorescence signal for three wild boar samples (Germany Bad Kissingen and Perleberg, and Europe) obtained with assay_Chr1*D*_ was not in accordance with their genotype and therefore caused by cross-reactivity. The majority of domestic pig individuals was found to be homozygous for the T allele. However, six out of eight Turopolje individuals were found to show the heterozygous C/T genotype, explaining why an increase in the fluorescence signal was obtained with both assay_Chr1*D*_ and assay_Chr1*W*_. The high frequency of the heterozygous C/T genotype in Turopolje, a domestic pig breed from Croatia, is in accordance with a very recent paper of the research group of Fontanesi. By genotyping 47 Turopolje individuals, the frequency of the T and the C allele were reported to be 0.57 and 0.43, respectively^19^.

Our results demonstrate that neither the two singleplex assays targeting SNP rs81416363 nor the duplex assay targeting SNP g.299084751 C > T alone allow the unambiguous discrimination of wild boar and domestic pig. However, by taking the results of the two singleplex assays and the duplex assay into consideration, the discrimination power can be drastically increased. Our results demonstrate that if the two assays for the same subspecies (e.g. assay_Chr9*D*_ and assay_Chr1*D*_) lead to the identical classification (in the given example domestic pig), and the results obtained with the two assays for the other subspecies (in the given example assay_Chr9*W*_ and assay_Chr1*W*_) are ambiguous, the probability of misclassification is low if one relies on the results that are identical. Following this strategy, 86 (91.5%) of 94 individuals could be assigned correctly.

## Materials and Methods

### Samples

64 domestic pig samples, comprising 14 different breeds and 6 cross-breeds (Suppl. Table 5), were mainly collected in Austria and Germany. 30 wild boar samples derived from Austria, Germany, Romania, USA, Estonia and Europe (exact origin unknown) (Suppl. Table 5). Samples of animal (Suppl. Table 5) and plant species (Suppl. Table 6) used for cross-reactivity tests were obtained from local meat and spice suppliers, the University of Veterinary Medicine, the Research Institute of Wildlife Ecology (both Vienna, Austria) and the Wildpark Ernstbrunn (Ernstbrunn, Austria).

### Sample preparation and DNA isolation

Aliquots of 1–2 g were weighed out and incubated with 10 mL CTAB extraction solution (2% (w/v) CTAB, 1.4 M NaCl, 0.1 M Tris, 0.02 M EDTA, adjusted to pH 8.0 with 4 M HCl, autoclaved) and 80 µL proteinase K solution at 50 °C (Unihood 750, Uniequip, Martinsried, Germany) under shaking (Intelli-Mixer RM-2L, LTF Labortechnik, Wasserburg, Germany) for at least 4 hours. DNA was isolated as described previously^20^ at least twice from one lysis batch. DNA concentration and purity were determined with a QIAxpert spectrophotometer (Qiagen, Hilden, Germany). DNA isolates were stored at −20 °C.

### Primers and probes

Primers and probes were designed using Primer Express 3.0 (Applied Biosystems). Primers were synthesised by Sigma Aldrich (Darmstadt, Germany), probes by Eurogentec (Seraing, Belgium). The sequences are given in Suppl. Table 1.

### Real-time PCR

Unless otherwise indicated, real-time PCR was performed on the Rotor Gene Q cycler (Qiagen) with a reaction mix consisting of 12.5 µL of QuantiTect Multiplex PCR NoROX Master Mix (Qiagen), 2.5 µL of ultrapure water, 5 µL of 5x primer/probe mix and 5 µL of isolated DNA (5–20 ng/µL). The standard temperature program included a denaturation step at 95 °C for 15 min, followed by 40 cycles at 94 °C for 1 min and 60 °C for 1 min.

### Optimisation of primer and probe concentrations

All optimisation experiments were performed with DNA isolates (10 ng/µL) from wild boar, domestic pig and roe deer. The primer and probe concentrations tested are summarised in Suppl. Table 4.

### Specificity of assays

For cross-reactivity tests, DNA isolates from 23 animal species (10 ng/µL) and 50 plant species (20 ng/µL) were used. The species are listed in Suppl. Tables 5 and 6, respectively.

### Use of other commercial master mixes and another real-time PCR system

Cross-reactivity tests were extended by analysing DNA isolates (10 ng/µL) from the 23 animal species (Suppl. Table 5) with four commercial master mixes, the GoTaq® Probe qPCR Master Mix (Promega), the PerfeCTa® qPCR ToughMix^TM^, Low ROX^TM^ (Quanta Biosciences), the Takyon^TM^ No Rox Probe MasterMix dTTP Blue (Eurogentec) and the TaqMan® Universal PCR Master Mix (Applied Biosystems). The temperature protocols are summarised in Suppl. Table 7.

### Working range, linear range and amplification efficiency

The working range of assay_Chr9*W*_ and assay_Chr1*W*_ were determined by analysing a wild boar DNA isolate (211 ng/µL) and serial dilutions thereof in water (1:2–1:524,288). For assay_Chr9*D*_ and assay_Chr1_, two DNA isolates (158 ng/ mL and 160 ng/µL, serial dilutions 1:2–1:524,288 in water) of domestic pig were used. The linear range was determined by analysing a wild boar DNA isolate and a domestic pig DNA isolate (20 ng/µL, serially diluted 1:2–1:16,384 in herring sperm DNA (20 ng/µL)), respectively.

### Determination of LOD

LOD was determined as recommended by the European Network of GMO Laboratories^17^. For the determination of the LODs of assay_Chr9*W*_ and assay_Chr1*W*_, a DNA mixture (5 ng/µL) containing 1% (w/w) wild boar and 99% (w/w) herring sperm DNA was serially diluted in herring sperm DNA (5 ng/µL) to obtain mixtures containing 0.5%, 0.4%, 0.3%, 0.2%, 0.1%, 0.05% or 0.01% wild boar. To determine the LOD of assay_Chr9*D*_, a DNA mixture containing 5% (w/w) domestic pig DNA and 95% (w/w) herring sperm DNA and the following serial dilutions thereof in herring sperm DNA (5 ng/µL) were analysed: 4%, 3%, 2%, 1%, 0.5%, 0.25% and 0.1%. The LOD of assay_Chr1*D*_ was determined by analysing a DNA mixture containing 1% (w/w) domestic pig DNA and 99% (w/w) herring sperm DNA and the following serial dilutions thereof in herring sperm DNA (5 ng/µL): 0.5%, 0.4%, 0.3%, 0.2%, 0.1%, 0.05% and 0.01%. The LOD was defined as the lowest concentration that was amplified in at least 19 out of 20 replicates with a Ct value that was below the Ct value obtained for cross-reacting species.

In addition, LOD of assay_Chr9*W*_ and assay_Chr1*W*_ was determined in domestic pig DNA as background DNA and of assay_Chr9*D*_ and assay_Chr1*D*_ in wild boar DNA as background DNA. DNA mixtures (5 ng/µL) containing 3%, 2%, 1%, 0.5%, 0.4%, 0.3%, 0.2% or 0.1% (w/w) wild boar DNA in domestic pig DNA were analysed using assay_Chr9*W*_. DNA mixtures (5 ng/µL) containing 5%, 4%, 3%, 2%, 1%, 0.5%, 0.25% or 0.1% (w/w) domestic pig DNA in wild boar DNA were analysed by assay_Chr9*D*_. DNA mixtures (5 ng/µL) containing either 20%, 10%, 5%, 4%, 3%, 2%, 1%, 0.75% and 0.5% (w/w) wild boar DNA in domestic pig or 40%, 20%, 10%, 5%, 4%, 3%, 2%, 1%, 0.5%, 0.25% and 0.1% (w/w) domestic pig DNA in wild boar DNA were analysed by assay_Chr1_.

### Investigation of signal suppression

Signal suppression, observed for assay_Chr1*D*_ and assay_Chr1*W*_, was investigated by analysing a DNA mixture (20 ng/µL) containing 25% (w/w) target subspecies DNA in non-target subspecies DNA as background and serial dilutions (1:4 to 1:1,024) thereof in water. The positive control contained 100% (w/w) target subspecies DNA (5 ng/µL) and had the same target subspecies DNA concentration as the 1:4 diluted standard. The extent of signal suppression was evaluated by comparing the maximum ΔRn values.

### Repeatability

The repeatability of the assays was investigated by analysing a meat extract mixture (20 ng/µL) consisting of 30% (w/w) wild boar and 70% (w/w) domestic pig and serial dilutions (1:4; 1:16; 1:64; 1:256; 1:1,024 in water) thereof in duplicates five times on four different days.

### Qualitative analysis

For qualitative analysis, all DNA isolates were diluted in water to a DNA concentration of 10 ng/µL. To each qualitative PCR plate a positive control (10 ng/µL) containing DNA of the target pig subspecies in the same concentration as the LOD was added, with the exception of assay_Chr9*D*_. The positive control for assay_Chr9*D*_ and assay_Chr9*W*_ was a DNA mixture containing 0.2% (w/w) wild boar DNA, 0.5% (w/w) domestic pig DNA and 99.3% (w/w) herring sperm DNA. For assay_Chr1_, a DNA mixture containing 2% (w/w) wild boar DNA, 5% (w/w) domestic pig DNA and 93% (w/w) herring sperm DNA was used.

### High resolution melting (HRM) analysis

Primers (fw: 5′ GTAAGAAAATCTTAACCTAGCAAATGGGT 3′; rv: 5′ CCAGGGAGTTTTTTGTTCTTTCA 3′) targeting SNP rs81416363 (g.118314929 A > G) on chromosome 9, generating a 75 bp long amplicon, were designed in-house. For the analysis of SNP g.299084751 C > T on chromosome 1, the real-time PCR primers (fw: 5′ CCTGGGAACAGGGCTTCA 3′; rv: 5′ AAGCTCACCTGGAGGACAGTGT 3′), generating a 67 bp long amplicon, were used. PCR and subsequent HRM analysis was carried out using a Rotor-Gene Q (Qiagen) thermocycler and the EpiTect HRM PCR Kit (Qiagen). PCR-HRM was carried out in a total volume of 20 µL. The reaction mixture consisted of 10 µL EpiTect HRM PCR Kit (Qiagen), 0.2 µM of each primer, 7.2 µL RNase-free water and 2 µL DNA extract (5 ng/µL). The PCR temperature program consisted of an initial denaturation at 95 °C for 5 min, followed by cycling (50 × (94 °C for 10 s; 60 °C for 20 s, 72 °C for 20 s)) and final elongation at 72 °C for 10 min. After denaturation at 95 °C for 1 min and hybridization at 40 °C for 1 min, HRM was performed by increasing the temperature from 65 °C to 95 °C in 0.1 °C increments per 2 s. Data was analysed using the Rotor-Gene Q Series Software 2.3.1 (Qiagen).

## Supplementary information

Supplementary Info
