# Supplementary material for: Differentiation between wild boar and domestic pig in food by targeting two gene loci by real-time PCR
Source: Sci Rep. 2019 Jun 25;9:9221. doi: 10.1038/s41598-019-45564-7 (PMC6593100; doi:10.1038/s41598-019-45564-7)
Supplement: Supplementary file 1 — Supplementary Info [file 41598_2019_45564_MOESM1_ESM.docx]

**Differentiation between wild boar and domestic pig in food by targeting two gene loci by real-time PCR**

Maria Kaltenbrunner^1,2^, Walter Mayer^1^, Kirsten Kerkhoff^3^, Rita Epp^3^, Hermann Rüggeberg^3^, Rupert Hochegger^1^ and Margit Cichna-Markl^2,*^

^1^Austrian Agency for Health and Food Safety, Institute for Food Safety Vienna, Department of Molecular Biology and Microbiology, Spargelfeldstraße 191, 1220 Vienna, Austria

^2^Department of Analytical Chemistry, Faculty of Chemistry, University of Vienna, Währinger Straße 38, 1090 Vienna, Austria

^3^Impetus GmbH & Co. Bioscience KG, Fischkai 1, 27572 Bremerhaven, Germany

***Corresponding author**

Tel: +43-1-4277-52374

Fax: +43-1-4277-9523

E-mail: [margit.cichna@univie.ac.at](mailto:margit.cichna@univie.ac.at)

**The co-authors e-mail addresses:**

Maria Kaltenbrunner: [maria.kaltenbrunner@ages.at](mailto:maria.kaltenbrunner@ages.at)

Walter Mayer: [walter.mayer@ages.at](mailto:walter.mayer@ages.at)

Kirsten Kerkhoff: [k.kerkhoff@impetus-bioscience.de](mailto:k.kerkhoff@impetus-bioscience.de)

Rita Epp: [r.epp@impetus-bioscience.de](mailto:r.epp@impetus-bioscience.de)

Hermann Rüggeberg: [h.rueggeberg@impetus-bioscience.de](mailto:h.rueggeberg@impetus-bioscience.de)

Rupert Hochegger: [rupert.hochegger@ages.at](mailto:rupert.hochegger@ages.at)

**Supplementary Table 1:** Primer and probe sequences tested in this study.

| **Chromosome / gene / SNP** | **System** |  | **Primer/probe sequence (5'-3')** | **Length (nt)** | **Amplicon (nt)** |
| --- | --- | --- | --- | --- | --- |
| Chromosome 1 / | Chr1 a | Chr1 f1 | TTGAAGCTCACCTGGAGGACA | 21 | 61 |
| *NR6A1* (AP009124) |  | Chr1 r1 | AGGGCTTCAGAGAGCAACCAG | 21 |  |
|  |  | Chr1 p1*_W_* | ***FAM-*** TGGAGCCC**G**GTGAG ***- MGBNFQ*** | 14 |  |
|  | Chr1 b | Chr1 f2 | TGGGAACAGGGCTTCAGAGA | 20 | 65 |
|  |  | Chr1 r2 | AAGCTCACCTGGAGGACAGTGT | 22 |  |
|  |  | Chr1 p2*_W_* | ***YY-*** AGCTCCTCAC**C**GGG ***-MGBNFQ*** | 14 |  |
|  |  | Chr1 p1*_D_* | ***FAM-*** CCAGCTCCTCAC**T**GG ***-MGBNFQ*** | 15 |  |
|  | Chr1 c | Chr1 f2 | TGGGAACAGGGCTTCAGAGA | 20 | 65 |
|  |  | Chr1 r2 | AAGCTCACCTGGAGGACAGTGT | 22 |  |
|  |  | Chr1 p2*_W_* | ***YY-*** AGCTCCTCAC**C**GGG ***-MGBNFQ*** | 14 |  |
|  | Chr1 d | Chr1 f3 | CCTGGGAACAGGGCTTCAG | 19 | 67 |
|  |  | Chr1 r2 | AAGCTCACCTGGAGGACAGTGT | 22 |  |
|  |  | Chr1 p2*_W_* | ***YY-*** AGCTCCTCAC**C**GGG ***-MGBNFQ*** | 14 |  |
|  | Chr1 e | Chr1 f4 | CCTGGGAACAGGGCTTCA | 18 | 67 |
|  |  | Chr1 r2 | AAGCTCACCTGGAGGACAGTGT | 22 |  |
|  |  | Chr1 p2*_W_* | ***YY-*** AGCTCCTCAC**C**GGG ***-MGBNFQ*** | 14 |  |
|  | Chr1 f | Chr1 f2 | TGGGAACAGGGCTTCAGAGA | 20 | 67 |
|  |  | Chr1 r3 | TGAAGCTCACCTGGAGGACAGT | 22 |  |
|  |  | Chr1 p2*_W_* | ***YY-*** AGCTCCTCAC**C**GGG ***-MGBNFQ*** | 14 |  |
|  | Chr1 g | Chr1 f3 | CCTGGGAACAGGGCTTCAG | 19 | 69 |
|  |  | Chr1 r3 | TGAAGCTCACCTGGAGGACAGT | 22 |  |
|  |  | Chr1 p2*_W_* | ***YY-*** AGCTCCTCAC**C**GGG ***-MGBNFQ*** | 14 |  |
|  | Chr1 h | Chr1 f4 | CCTGGGAACAGGGCTTCA | 18 | 69 |
|  |  | Chr1 r3 | TGAAGCTCACCTGGAGGACAGT | 22 |  |
|  |  | Chr1 p2*_W_* | ***YY-*** AGCTCCTCAC**C**GGG ***-MGBNFQ*** | 14 |  |
|  | Chr1 i | Chr1 f2 | TGGGAACAGGGCTTCAGAGA | 20 | 65 |
|  |  | Chr1 r2 | AAGCTCACCTGGAGGACAGTGT | 22 |  |
|  |  | Chr1 p3*_W_* | ***YY-*** CTCCTCAC**C**GGGCT ***-MGBNFQ*** | 14 |  |
|  | Chr1 j | Chr1 f3 | CCTGGGAACAGGGCTTCAG | 19 | 67 |
|  |  | Chr1 r2 | AAGCTCACCTGGAGGACAGTGT | 22 |  |
|  |  | Chr1 p3*_W_* | ***YY-*** CTCCTCAC**C**GGGCT ***-MGBNFQ*** | 14 |  |
|  | Chr1 k | Chr1 f4 | CCTGGGAACAGGGCTTCA | 18 | 67 |
|  |  | Chr1 r2 | AAGCTCACCTGGAGGACAGTGT | 22 |  |
|  |  | Chr1 p3*_W_* | ***YY-*** CTCCTCAC**C**GGGCT ***-MGBNFQ*** | 14 |  |
|  | Chr1 l | Chr1 f2 | TGGGAACAGGGCTTCAGAGA | 20 | 67 |
|  |  | Chr1 r3 | TGAAGCTCACCTGGAGGACAGT | 22 |  |
|  |  | Chr1 p3*_W_* | ***YY-*** CTCCTCAC**C**GGGCT ***-MGBNFQ*** | 14 |  |
|  | Chr1 m | Chr1 f3 | CCTGGGAACAGGGCTTCAG | 19 | 69 |
|  |  | Chr1 r3 | TGAAGCTCACCTGGAGGACAGT | 22 |  |
|  |  | Chr1 p3*_W_* | ***YY-*** CTCCTCAC**C**GGGCT ***-MGBNFQ*** | 14 |  |
|  | Chr1 n | Chr1 f4 | CCTGGGAACAGGGCTTCA | 18 | 69 |
|  |  | Chr1 r3 | TGAAGCTCACCTGGAGGACAGT | 22 |  |
|  |  | Chr1 p3*_W_* | ***YY-*** CTCCTCAC**C**GGGCT ***-MGBNFQ*** | 14 |  |
|  | Chr1 o | Chr1 f2 | TGGGAACAGGGCTTCAGAGA | 20 | 65 |
|  |  | Chr1 r2 | AAGCTCACCTGGAGGACAGTGT | 22 |  |
|  |  | Chr1 p4*_W_* | ***YY-*** CTCAC**C**GGGCTC ***-MGBNFQ*** | 12 |  |
|  | Chr1 p | Chr1 f3 | CCTGGGAACAGGGCTTCAG | 19 | 67 |
|  |  | Chr1 r2 | AAGCTCACCTGGAGGACAGTGT | 22 |  |
|  |  | Chr1 p4*_W_* | ***YY-*** CTCAC**C**GGGCTC ***-MGBNFQ*** | 12 |  |
|  | Chr1 q | Chr1 f4 | CCTGGGAACAGGGCTTCA | 18 | 67 |
|  |  | Chr1 r2 | AAGCTCACCTGGAGGACAGTGT | 22 |  |
|  |  | Chr1 p4*_W_* | ***YY-*** CTCAC**C**GGGCTC ***-MGBNFQ*** | 12 |  |
|  | Chr1 r | Chr1 f2 | TGGGAACAGGGCTTCAGAGA | 20 | 67 |
|  |  | Chr1 r3 | TGAAGCTCACCTGGAGGACAGT | 22 |  |
|  |  | Chr1 p4*_W_* | ***YY-*** CTCAC**C**GGGCTC ***-MGBNFQ*** | 12 |  |
|  | Chr1 s | Chr1 f3 | CCTGGGAACAGGGCTTCAG | 19 | 69 |
|  |  | Chr1 r3 | TGAAGCTCACCTGGAGGACAGT | 22 |  |
|  |  | Chr1 p4*_W_* | ***YY-*** CTCAC**C**GGGCTC ***-MGBNFQ*** | 12 |  |
|  | Chr1 t | Chr1 f4 | CCTGGGAACAGGGCTTCA | 18 | 69 |
|  |  | Chr1 r3 | TGAAGCTCACCTGGAGGACAGT | 22 |  |
|  |  | Chr1 p4*_W_* | ***YY-*** CTCAC**C**GGGCTC ***-MGBNFQ*** | 12 |  |
|  | Chr1 u | Chr1 f4 | CCTGGGAACAGGGCTTCA | 18 | 67 |
|  |  | Chr1 r2 | AAGCTCACCTGGAGGACAGTGT | 22 |  |
|  |  | Chr1 p4*_W_* | ***YY-*** CTCAC**C**GGGCTC ***-MGBNFQ*** | 12 |  |
|  |  | Chr1 p1*_D_* | ***FAM-*** CCAGCTCCTCAC**T**GG ***-MGBNFQ*** | 15 |  |
| f… forward primer, r… reverse primer, p… probe, FAM… 6-carboxyfluorescein (fluorescent dye), YY…yakima yellow (fluorescent dye), *W*… wild boar, *D*… domestic pig, MGBNFQ… minor groove binding non-fluorescent quencher | | | | | |

| **Chromosome / gene / SNP** | **System** |  | **Primer/probe sequence (5'-3')** | **Length (nt)** | **Amplicon (nt)** |
| --- | --- | --- | --- | --- | --- |
| Chromosome 5 / | Chr5 a | Chr5 f1 | GCCAGCTGCTCAGCCGA | 17 | 61 |
| Intergenic / |  | Chr5 r1*_W_* | TATAGGTCTGGGCCCACCATA**C**T | 23 |  |
| rs80864596 |  | Chr5 p1 | ***FAM-***CCTGGGAGTAAAGCTAA***-MGBNFQ*** | 17 |  |
|  | Chr5 b | Chr5 f1 | GCCAGCTGCTCAGCCGA | 17 | 57 |
|  |  | Chr5 r2*_W_* | GGTCTGGGCCCACCATT**C**T | 19 |  |
|  |  | Chr5 p1 | ***FAM-*** CCTGGGAGTAAAGCTAA ***-MGBNFQ*** | 17 |  |
|  | Chr5 c | Chr5 f1 | GCCAGCTGCTCAGCCGA | 17 | 56 |
|  |  | Chr5 r3*_W_* | GTCTGGGCCCACCATC**C**T | 18 |  |
|  |  | Chr5 p1 | ***FAM-*** CCTGGGAGTAAAGCTAA ***-MGBNFQ*** | 17 |  |
|  | Chr5 d | Chr5 f1 | GCCAGCTGCTCAGCCGA | 17 | 55 |
|  |  | Chr5 r4*_W_* | TCTGGGCCCACCATG**C**T | 17 |  |
|  |  | Chr5 p1 | ***FAM-*** CCTGGGAGTAAAGCTAA ***-MGBNFQ*** | 17 |  |
| Chromosome 9 / | Chr9 a | Chr9 f1*_W_* | CTCACAGGTGATGTGACTGC**G**T | 22 | 79 |
| intergenic / |  | Chr9 r1 | GGCCCATCCTAGTGAGAAACC | 21 |  |
| rs81416363 |  | Chr9 p1 | ***FAM-*** CCTGGCCACATGAGA ***-MGBNFQ*** | 15 |  |
|  | Chr9 b | Chr9 f2*_W_* | CTCACAGGTGATGTGACTGG**G**T | 22 | 79 |
|  |  | Chr9 r1 | GGCCCATCCTAGTGAGAAACC | 21 |  |
|  |  | Chr9 p1 | ***FAM-*** CCTGGCCACATGAGA ***-MGBNFQ*** | 15 |  |
|  | Chr9 c | Chr9 f3*_W_* | TGTCTCACAGGTGATGTGACTGT**G**T | 25 | 82 |
|  |  | Chr9 r1 | GGCCCATCCTAGTGAGAAACC | 21 |  |
|  |  | Chr9 p1 | ***FAM-*** CCTGGCCACATGAGA ***-MGBNFQ*** | 15 |  |
|  | Chr9 d | Chr9 f4*_W_* | TGTCTCACAGGTGATGTGACTGA**G**T | 25 | 82 |
|  |  | Chr9 r1 | GGCCCATCCTAGTGAGAAACC | 21 |  |
|  |  | Chr9 p1 | ***FAM-*** CCTGGCCACATGAGA ***-MGBNFQ*** | 15 |  |
|  | Chr9 e | Chr9 f5*_W_* | CTCACAGGTGATGTGAGTGC**G**T | 22 | 79 |
|  |  | Chr9 r1 | GGCCCATCCTAGTGAGAAACC | 21 |  |
|  |  | Chr9 p1 | ***FAM-*** CCTGGCCACATGAGA ***-MGBNFQ*** | 15 |  |
|  | Chr9 f | Chr9 f6*_W_* | TCTCACAGGTGATGTGATTGC**G**T | 23 | 80 |
|  |  | Chr9 r1 | GGCCCATCCTAGTGAGAAACC | 21 |  |
|  |  | Chr9 p1 | ***FAM-*** CCTGGCCACATGAGA ***-MGBNFQ*** | 15 |  |
|  | Chr9 g | Chr9 f7*_W_* | CTCACAGGTGATGTGAATGC**G**T | 22 | 79 |
|  |  | Chr9 r1 | GGCCCATCCTAGTGAGAAACC | 21 |  |
|  |  | Chr9 p1 | ***FAM-*** CCTGGCCACATGAGA ***-MGBNFQ*** | 15 |  |
|  | Chr9 h | Chr9 f8 | GTAAGAAAATCTTAACCTAGCAAATGGGT | 29 | 74 |
|  |  | Chr9 r2*_W_* | CAGGGAGTTTTTTGTTCTTTCA**C**G | 24 |  |
|  |  | Chr9 p2 | ***FAM-*** CTCACAGGTGATGTGACT ***-MGBNFQ*** | 18 |  |
|  | Chr9 i | Chr9 f8 | GTAAGAAAATCTTAACCTAGCAAATGGGT | 29 | 74 |
|  |  | Chr9 r3*_W_* | CAGGGAGTTTTTTGTTCTTTCT**C**G | 24 |  |
|  |  | Chr9 p2 | ***FAM-*** CTCACAGGTGATGTGACT ***-MGBNFQ*** | 18 |  |
|  | Chr9 j | Chr9 f8 | GTAAGAAAATCTTAACCTAGCAAATGGGT | 29 | 72 |
|  |  | Chr9 r4*_W_* | GGGAGTTTTTTGTTCTTTCC**C**G | 22 |  |
|  |  | Chr9 p2 | ***FAM-*** CTCACAGGTGATGTGACT ***-MGBNFQ*** | 18 |  |
|  | Chr9 k | Chr9 f8 | GTAAGAAAATCTTAACCTAGCAAATGGGT | 29 | 72 |
|  |  | Chr9 r5*_W_* | GGGAGTTTTTTGTTCTTTCG**C**G | 22 |  |
|  |  | Chr9 p2 | ***FAM-*** CTCACAGGTGATGTGACT ***-MGBNFQ*** | 18 |  |
|  | Chr9 l | Chr9 f8 | GTAAGAAAATCTTAACCTAGCAAATGGGT | 29 | 74 |
|  |  | Chr9 r6*_W_* | CAGGGAGTTTTTTGTTCTATCA**C**G | 24 |  |
|  |  | Chr9 p2 | ***FAM-*** CTCACAGGTGATGTGACT ***-MGBNFQ*** | 18 |  |
|  | Chr9 m | Chr9 f8 | GTAAGAAAATCTTAACCTAGCAAATGGGT | 29 | 74 |
|  |  | Chr9 r7*_W_* | CAGGGAGTTTTTTGTTCTCTCA**C**G | 24 |  |
|  |  | Chr9 p2 | ***FAM-*** CTCACAGGTGATGTGACT ***-MGBNFQ*** | 18 |  |
|  | Chr9 n | Chr9 f8 | GTAAGAAAATCTTAACCTAGCAAATGGGT | 29 | 73 |
|  |  | Chr9 r8*_W_* | AGGGAGTTTTTTGTTCTGTCA**C**G | 23 |  |
|  |  | Chr9 p2 | ***FAM-*** CTCACAGGTGATGTGACT ***-MGBNFQ*** | 18 |  |
|  | Chr9 o | Chr9 f8 | GTAAGAAAATCTTAACCTAGCAAATGGGT | 29 | 72 |
|  |  | Chr9 r9*_W_* | GGGAGTTTTTTGTTCTTTGC**C**G | 22 |  |
|  |  | Chr9 p2 | ***FAM-*** CTCACAGGTGATGTGACT ***-MGBNFQ*** | 18 |  |
|  | Chr9 p | Chr9 f8 | GTAAGAAAATCTTAACCTAGCAAATGGGT | 29 | 74 |
|  |  | Chr9 r10*_W_* | CAGGGAGTTTTTTGTTCTTTAC**C**G | 24 |  |
|  |  | Chr9 p2 | ***FAM-*** CTCACAGGTGATGTGACT ***-MGBNFQ*** | 18 |  |
|  | Chr9 q | Chr9 f8 | GTAAGAAAATCTTAACCTAGCAAATGGGT | 29 | 73 |
|  |  | Chr9 r11*_W_* | AGGGAGTTTTTTGTTCTTTTC**C**G | 23 |  |
|  |  | Chr9 p2 | ***FAM-*** CTCACAGGTGATGTGACT ***-MGBNFQ*** | 18 |  |
| f… forward primer, r… reverse primer, p… probe, FAM… 6-carboxyfluorescein (fluorescent dye), YY…yakima yellow (fluorescent dye), *W*… wild boar, *D*… domestic pig, MGBNFQ… minor groove binding non-fluorescent quencher | | | | | |

| **Chromosome / gene / SNP** | **System** |  | **Primer/probe sequence (5'-3')** | **Length (nt)** | **Amplicon (nt)** |
| --- | --- | --- | --- | --- | --- |
| Chromosome 9 / | Chr9 r | Chr9 f8 | GTAAGAAAATCTTAACCTAGCAAATGGGT | 29 | 73 |
| intergenic / |  | Chr9 r12*_W_* | AGGGAGTTTTTTGTTCTTACC**C**G | 23 |  |
| rs81416363 |  | Chr9 p2 | ***FAM-*** CTCACAGGTGATGTGACT ***-MGBNFQ*** | 18 |  |
|  | Chr9 s | Chr9 f8 | GTAAGAAAATCTTAACCTAGCAAATGGGT | 29 | 71 |
|  |  | Chr9 r13*_W_* | GGAGTTTTTTGTTCTTCCC**C**G | 21 |  |
|  |  | Chr9 p2 | ***FAM-*** CTCACAGGTGATGTGACT ***-MGBNFQ*** | 18 |  |
|  | Chr9 t | Chr9 f8 | GTAAGAAAATCTTAACCTAGCAAATGGGT | 29 | 71 |
|  |  | Chr9 r14*_W_* | GGAGTTTTTTGTTCTTGCC**C**G | 21 |  |
|  |  | Chr9 p2 | ***FAM-*** CTCACAGGTGATGTGACT ***-MGBNFQ*** | 18 |  |
|  | Chr9 u | Chr9 f8 | GTAAGAAAATCTTAACCTAGCAAATGGGT | 29 | 75 |
|  |  | Chr9 r1*_D_* | CCAGGGAGTTTTTTGTTCTTTCA**T**G | 25 |  |
|  |  | Chr9 p2 | ***FAM-*** CTCACAGGTGATGTGACT ***-MGBNFQ*** | 18 |  |
|  | Chr9 v | Chr9 f8 | GTAAGAAAATCTTAACCTAGCAAATGGGT | 29 | 75 |
|  |  | Chr9 r2*_D_* | CCAGGGAGTTTTTTGTTCTTTCT**T**G | 25 |  |
|  |  | Chr9 p2 | ***FAM-*** CTCACAGGTGATGTGACT ***-MGBNFQ*** | 18 |  |
|  | Chr9 w | Chr9 f8 | GTAAGAAAATCTTAACCTAGCAAATGGGT | 29 | 74 |
|  |  | Chr9 r3*_D_* | CAGGGAGTTTTTTGTTCTTTCC**T**G | 24 |  |
|  |  | Chr9 p2 | ***FAM-*** CTCACAGGTGATGTGACT ***-MGBNFQ*** | 18 |  |
|  | Chr9 x | Chr9 f8 | GTAAGAAAATCTTAACCTAGCAAATGGGT | 29 | 74 |
|  |  | Chr9 r4*_D_* | CAGGGAGTTTTTTGTTCTTTCG**T**G | 24 |  |
|  |  | Chr9 p2 | ***FAM-*** CTCACAGGTGATGTGACT ***-MGBNFQ*** | 18 |  |
|  | Chr9 y | Chr9 f8 | GTAAGAAAATCTTAACCTAGCAAATGGGT | 29 | 75 |
|  |  | Chr9 r5*_D_* | CCAGGGAGTTTTTTGTTCTTACT**T**G | 25 |  |
|  |  | Chr9 p2 | ***FAM-*** CTCACAGGTGATGTGACT ***-MGBNFQ*** | 18 |  |
|  | Chr9 z | Chr9 f8 | GTAAGAAAATCTTAACCTAGCAAATGGGT | 29 | 74 |
|  |  | Chr9 r6*_D_* | CAGGGAGTTTTTTGTTCTTCCT**T**G | 24 |  |
|  |  | Chr9 p2 | ***FAM-*** CTCACAGGTGATGTGACT ***-MGBNFQ*** | 18 |  |
|  | Chr9 aa | Chr9 f8 | GTAAGAAAATCTTAACCTAGCAAATGGGT | 29 | 74 |
|  |  | Chr9 r7*_D_* | CAGGGAGTTTTTTGTTCTTGCT**T**G | 24 |  |
|  |  | Chr9 p2 | ***FAM-*** CTCACAGGTGATGTGACT ***-MGBNFQ*** | 18 |  |
|  | Chr9 ab | Chr9 f8 | GTAAGAAAATCTTAACCTAGCAAATGGGT | 29 | 75 |
|  |  | Chr9 r8*_D_* | CCAGGGAGTTTTTTGTTCTTACC**T**G | 25 |  |
|  |  | Chr9 p2 | ***FAM-*** CTCACAGGTGATGTGACT ***-MGBNFQ*** | 18 |  |
|  | Chr9 ac | Chr9 f8 | GTAAGAAAATCTTAACCTAGCAAATGGGT | 29 | 73 |
|  |  | Chr9 r9*_D_* | AGGGAGTTTTTTGTTCTTCCC**T**G | 23 |  |
|  |  | Chr9 p2 | ***FAM-*** CTCACAGGTGATGTGACT ***-MGBNFQ*** | 18 |  |
|  | Chr9 ad | Chr9 f8 | GTAAGAAAATCTTAACCTAGCAAATGGGT | 29 | 73 |
|  |  | Chr9 r10*_D_* | AGGGAGTTTTTTGTTCTTGCC**T**G | 23 |  |
|  |  | Chr9 p2 | ***FAM-*** CTCACAGGTGATGTGACT ***-MGBNFQ*** | 18 |  |
|  | Chr9 ae | Chr9 f8 | GTAAGAAAATCTTAACCTAGCAAATGGGT | 29 | 74 |
|  |  | Chr9 r11*_D_* | CAGGGAGTTTTTTGTTCTTACGTG | 24 |  |
|  |  | Chr9 p2 | ***FAM-*** CTCACAGGTGATGTGACT ***-MGBNFQ*** | 18 |  |
|  | Chr9 af | Chr9 f8 | GTAAGAAAATCTTAACCTAGCAAATGGGT | 29 | 73 |
|  |  | Chr9 r12*_D_* | AGGGAGTTTTTTGTTCTTCCG**T**G | 23 |  |
|  |  | Chr9 p2 | ***FAM-*** CTCACAGGTGATGTGACT ***-MGBNFQ*** | 18 |  |
|  | Chr9 ag | Chr9 f8 | GTAAGAAAATCTTAACCTAGCAAATGGGT | 29 | 72 |
|  |  | Chr9 r13*_D_* | GGGAGTTTTTTGTTCTTGCG**T**G | 22 |  |
|  |  | Chr9 p2 | ***FAM-*** CTCACAGGTGATGTGACT ***-MGBNFQ*** | 18 |  |
|  | Chr9 ah | Chr9 f8 | GTAAGAAAATCTTAACCTAGCAAATGGGT | 29 | 74 |
|  |  | Chr9 r14*_D_* | CAGGGAGTTTTTTGTTCTTTGT**T**G | 24 |  |
|  |  | Chr9 p2 | ***FAM-*** CTCACAGGTGATGTGACT ***-MGBNFQ*** | 18 |  |
|  | Chr9 ai | Chr9 f8 | GTAAGAAAATCTTAACCTAGCAAATGGGT | 29 | 75 |
|  |  | Chr9 r15*_D_* | CCAGGGAGTTTTTTGTTCTTTAT**T**G | 25 |  |
|  |  | Chr9 p2 | ***FAM-*** CTCACAGGTGATGTGACT ***-MGBNFQ*** | 18 |  |
|  | Chr9 aj | Chr9 f8 | GTAAGAAAATCTTAACCTAGCAAATGGGT | 29 | 75 |
|  |  | Chr9 r16*_D_* | CCAGGGAGTTTTTTGTTCTTTTT**T**G | 25 |  |
|  |  | Chr9 p2 | ***FAM-*** CTCACAGGTGATGTGACT ***-MGBNFQ*** | 18 |  |
|  | Chr9 ak | Chr9 f8 | GTAAGAAAATCTTAACCTAGCAAATGGGT | 29 | 74 |
|  |  | Chr9 r17*_D_* | CAGGGAGTTTTTTGTTCTTGGT**T**G | 24 |  |
|  |  | Chr9 p2 | ***FAM-*** CTCACAGGTGATGTGACT ***-MGBNFQ*** | 18 |  |
|  | Chr9 al | Chr9 f8 | GTAAGAAAATCTTAACCTAGCAAATGGGT | 29 | 75 |
|  |  | Chr9 r18*_D_* | CCAGGGAGTTTTTTGTTCTTGAT**T**G | 25 |  |
|  |  | Chr9 p2 | ***FAM-*** CTCACAGGTGATGTGACT ***-MGBNFQ*** | 18 |  |
|  | Chr9 am | Chr9 f8 | GTAAGAAAATCTTAACCTAGCAAATGGGT | 29 | 74 |
|  |  | Chr9 r19*_D_* | CAGGGAGTTTTTTGTTCTTGTT**T**G | 24 |  |
|  |  | Chr9 p2 | ***FAM-*** CTCACAGGTGATGTGACT ***-MGBNFQ*** | 18 |  |
|  | Chr9 an | Chr9 f8 | GTAAGAAAATCTTAACCTAGCAAATGGGT | 29 | 73 |
|  |  | Chr9 r20*_D_* | AGGGAGTTTTTTGTTCATGGT**T**G | 23 |  |
|  |  | Chr9 p2 | ***FAM-*** CTCACAGGTGATGTGACT ***-MGBNFQ*** | 18 |  |
| f… forward primer, r… reverse primer, p… probe, FAM… 6-carboxyfluorescein (fluorescent dye), YY…yakima yellow (fluorescent dye), *W*… wild boar, *D*… domestic pig, MGBNFQ… minor groove binding non-fluorescent quencher | | | | | |

| **Chromosome / gene / SNP** | **System** |  | **Primer/probe sequence (5'-3')** | **Length (nt)** | **Amplicon (nt)** |
| --- | --- | --- | --- | --- | --- |
| Chromosome 9 / | Chr9 ao | Chr9 f8 | GTAAGAAAATCTTAACCTAGCAAATGGGT | 29 | 74 |
| intergenic / |  | Chr9 r21*_D_* | CAGGGAGTTTTTTGTTCCTGAT**T**G | 24 |  |
| rs81416363 |  | Chr9 p2 | ***FAM-*** CTCACAGGTGATGTGACT ***-MGBNFQ*** | 18 |  |
|  | Chr9 ap | Chr9 f8 | GTAAGAAAATCTTAACCTAGCAAATGGGT | 29 | 73 |
|  |  | Chr9 r22*_D_* | AGGGAGTTTTTTGTTCGTGTT**T**G | 23 |  |
|  |  | Chr9 p2 | ***FAM-*** CTCACAGGTGATGTGACT ***-MGBNFQ*** | 18 |  |
|  | Chr9 aq | Chr9 f8 | GTAAGAAAATCTTAACCTAGCAAATGGGT | 29 | 75 |
|  |  | Chr9 r23*_D_* | CCAGGGAGTTTTTTGTTCTAGCT**T**G | 25 |  |
|  |  | Chr9 p2 | ***FAM-*** CTCACAGGTGATGTGACT ***-MGBNFQ*** | 18 |  |
|  | Chr9 ar | Chr9 f8 | GTAAGAAAATCTTAACCTAGCAAATGGGT | 29 | 73 |
|  |  | Chr9 r24*_D_* | AGGGAGTTTTTTGTTCTCGCT**T**G | 23 |  |
|  |  | Chr9 p2 | ***FAM-*** CTCACAGGTGATGTGACT ***-MGBNFQ*** | 18 |  |
|  | Chr9 as | Chr9 f8 | GTAAGAAAATCTTAACCTAGCAAATGGGT | 29 | 73 |
|  |  | Chr9 r25*_D_* | AGGGAGTTTTTTGTTCTGGCT**T**G | 23 |  |
|  |  | Chr9 p2 | ***FAM-*** CTCACAGGTGATGTGACT ***-MGBNFQ*** | 18 |  |
|  | Chr9 at | Chr9 f8 | GTAAGAAAATCTTAACCTAGCAAATGGGT | 29 | 73 |
|  |  | Chr9 r26*_D_* | AGGGAGTTTTTTGTTCATGCT**T**G | 23 |  |
|  |  | Chr9 p2 | ***FAM-*** CTCACAGGTGATGTGACT ***-MGBNFQ*** | 18 |  |
|  | Chr9 au | Chr9 f8 | GTAAGAAAATCTTAACCTAGCAAATGGGT | 29 | 73 |
|  |  | Chr9 r27*_D_* | AGGGAGTTTTTTGTTCCTGCT**T**G | 23 |  |
|  |  | Chr9 p2 | ***FAM-*** CTCACAGGTGATGTGACT ***-MGBNFQ*** | 18 |  |
|  | Chr9 av | Chr9 f8 | GTAAGAAAATCTTAACCTAGCAAATGGGT | 29 | 72 |
|  |  | Chr9 r28*_D_* | GGGAGTTTTTTGTTCGTGCT**T**G | 22 |  |
|  |  | Chr9 p2 | ***FAM-*** CTCACAGGTGATGTGACT ***-MGBNFQ*** | 18 |  |
|  | Chr9 aw | Chr9 f8 | GTAAGAAAATCTTAACCTAGCAAATGGGT | 29 | 74 |
|  |  | Chr9 r29*_D_* | CAGGGAGTTTTTTGTACATGCT**T**G | 24 |  |
|  |  | Chr9 p2 | ***FAM-*** CTCACAGGTGATGTGACT ***-MGBNFQ*** | 18 |  |
|  | Chr9 ax | Chr9 f8 | GTAAGAAAATCTTAACCTAGCAAATGGGT | 29 | 72 |
|  |  | Chr9 r30*_D_* | GGGAGTTTTTTGTCCCTGCT**T**G | 22 |  |
|  |  | Chr9 p2 | ***FAM-*** CTCACAGGTGATGTGACT ***-MGBNFQ*** | 18 |  |
|  | Chr9 ay | Chr9 f8 | GTAAGAAAATCTTAACCTAGCAAATGGGT | 29 | 71 |
|  |  | Chr9 r31*_D_* | GGAGTTTTTTGTGCGTGCT**T**G | 21 |  |
|  |  | Chr9 p2 | ***FAM-*** CTCACAGGTGATGTGACT ***-MGBNFQ*** | 18 |  |
|  | Chr9 az | Chr9 f8 | GTAAGAAAATCTTAACCTAGCAAATGGGT | 29 | 75 |
|  |  | Chr9 r32*_D_* | CCAGGGAGTTTTTTATACATGCT**T**G | 25 |  |
|  |  | Chr9 p2 | ***FAM-*** CTCACAGGTGATGTGACT ***-MGBNFQ*** | 18 |  |
|  | Chr9 ba | Chr9 f8 | GTAAGAAAATCTTAACCTAGCAAATGGGT | 29 | 72 |
|  |  | Chr9 r33*_D_* | GGGAGTTTTTTCTCCCTGCT**T**G | 22 |  |
|  |  | Chr9 p2 | ***FAM-*** CTCACAGGTGATGTGACT ***-MGBNFQ*** | 18 |  |
|  | Chr9 bb | Chr9 f8 | GTAAGAAAATCTTAACCTAGCAAATGGGT | 29 | 71 |
|  |  | Chr9 r34*_D_* | GGAGTTTTTTTTGCGTGCT**T**G | 21 |  |
|  |  | Chr9 p2 | ***FAM-*** CTCACAGGTGATGTGACT ***-MGBNFQ*** | 18 |  |
| Chromosome 13 / | Chr13 a | Chr13 f1*_W_* | GTATGTATTCATTATTGAATGAAAACTTAACC**A**C | 34 | 96 |
| *Glycogen synthase kinase 3-beta* / |  | Chr13 r1 | CTAATTGGCAAGGAAAATAAATGAACTAG | 29 |  |
| rs80796712 |  | Chr13 p1 | ***FAM-*** ATTCATCCATAAGCTATGTGTTG ***-MGBNFQ*** | 23 |  |
|  | Chr13 b | Chr13 f2*_W_* | GTATGTATTCATTATTGAATGAAAACTTAACG**A**C | 34 | 96 |
|  |  | Chr13 r1 | CTAATTGGCAAGGAAAATAAATGAACTAG | 29 |  |
|  |  | Chr13 p1 | ***FAM-*** ATTCATCCATAAGCTATGTGTTG ***-MGBNFQ*** | 23 |  |
|  | Chr13 c | Chr13 f3*_W_* | TGTATGTATTCATTATTGAATGAAAACTTAACT**A**C | 35 | 97 |
|  |  | Chr13 r1 | CTAATTGGCAAGGAAAATAAATGAACTAG | 29 |  |
|  |  | Chr13 p1 | ***FAM-*** ATTCATCCATAAGCTATGTGTTG ***-MGBNFQ*** | 23 |  |
| f… forward primer, r… reverse primer, p… probe, FAM… 6-carboxyfluorescein (fluorescent dye), YY…yakima yellow (fluorescent dye), *W*… wild boar, *D*… domestic pig, MGBNFQ… minor groove binding non-fluorescent quencher | | | | | |

**Supplementary Table 2:** Results of preliminary selectivity tests (n = 2) for primer/probe systems targeting the *NR6A1* gene*.*

Primer/probe system Chr1a targeted the upper strand, primer/probe systems Chr1b - u the lower strand. All experiments were performed with the TaqMan® Universal PCR Master Mix (Applied Biosystems, Foster City, CA, USA) using forward primer/ reverse primer/ probe concentrations of 500 nM/ 500 nM/ 200 nM. DNA extracts were used at a concentration of 10 ng/µL. Primer/probe system Chr1a used probe p1, systems Chr1b – h probe p2, systems Chr1i – n probe p3 and systems Chr1o – t probe p4. Primer/probe systems Chr1a – t were tested as singleplex assays, primer/probe system Chr1t was additionally tested in combination with the domestic pig specific probe as a duplex assay (Chr1u).

| **System** | **Chr1a** | **Chr1b** | **Chr1c** | **Chr1d** | **Chr1e** | **Chr1f** | **Chr1g** | **Chr1h** | **Chr1i** | **Chr1j** | | **Chr1k** | | **Chr1l** | | **Chr1m** | | **Chr1n** | **Chr1o** | **Chr1p** | **Chr1q** |
| --- | --- | --- | --- | --- | --- | --- | --- | --- | --- | --- | --- | --- | --- | --- | --- | --- | --- | --- | --- | --- | --- |
| **Species** | **Ct** | **Ct** | **Ct** | **Ct** | **Ct** | **Ct** | **Ct** | **Ct** | **Ct** | **Ct** | | **Ct** | | **Ct** | | **Ct** | | **Ct** | **Ct** | **Ct** | **Ct** |
| Domestic pig |  |  | -^a^ | -^a^ | -^a^ | -^a^ | -^a^ | -^a^ | 33.04 | 34.19 | | 34.98 | | 33.67 | | 34.04 | | 35.23 | -^a^ | -^a^ | -^a^ |
| Iberian | 35.70 | -^a^ |  |  |  |  |  |  |  |  | |  | |  | |  | |  |  |  |  |
| Swabian-Hall | 36.33 | -^a^ |  |  |  |  |  |  |  |  | |  | |  | |  | |  |  |  |  |
| Duroc | 36.54 | -^a^ | -^a^ | -^a^ | -^a^ | -^a^ | -^a^ | -^a^ | 35.06 | 35.94 | | 36.65 | | 34.51 | | 35.46 | | 35.60 | 35.88 | -^a^ | -^a^ |
| Mangalica | 36.99 |  |  |  |  |  |  |  |  |  | |  | |  | |  | |  |  |  |  |
| Edelschwein | 36.26 |  |  |  |  |  |  |  |  |  | |  | |  | |  | |  |  |  |  |
| Landrace | 38.46 |  |  |  |  |  |  |  |  |  | |  | |  | |  | |  |  |  |  |
| Pietrain | 37.35 | -^a^ |  |  |  |  |  |  |  |  | |  | |  | |  | |  |  |  |  |
| Edelschwein x Landrace | 36.62 |  |  |  |  |  |  |  |  |  | |  | |  | |  | |  |  |  |  |
| Landrace x Edelschwein | 35.83 |  |  |  |  |  |  |  |  |  | |  | |  | |  | |  |  |  |  |
| Pietrain x Landrace + Edelschwein | 35.43 |  |  |  |  |  |  |  |  |  | |  | |  | |  | |  |  |  |  |
| Duroc x Landrace | 38.94 |  |  |  |  |  |  |  |  |  | |  | |  | |  | |  |  |  |  |
| Wild boar | 24.87 | 28.77 | 27.50 | 27.30 | 27.40 | 28.67 | 28.21 | 28.54 | 24.39 | 24.24 | | 24.18 | | 24.29 | | 24.28 | | 24.03 | 25.07 | 25.02 | 24.77 |
| **System** | **Chr1r** | **Chr1s** | **Chr1t** | **Chr1u*_W_*^b^** | **Chr1u*_D_*^b^** |  |  |  |  |  |  | |  | |  | |  | |  |  |  |
| **Species** | **Ct** | **Ct** | **Ct** | **Ct** | **Ct** |  |  |  |  |  |  | |  | |  | |  | |  |  |  |
| Domestic pig | -^a^ | -^a^ | -^a^ |  |  |  |  |  |  |  |  | |  | |  | |  | |  |  |  |
| Duroc | -^a^ | -^a^ | -^a^ |  |  |  |  |  |  |  |  | |  | |  | |  | |  |  |  |
| Edelschwein |  |  |  | -^a^ | 27.90 |  |  |  |  |  |  | |  | |  | |  | |  |  |  |
| Wild boar | 26.08 | 25.58 | 25.40 | 26.21 | -^a^ |  |  |  |  |  |  | |  | |  | |  | |  |  |  |
| ^a^ no increase in the fluorescence signal within 40 cycles, ^b^Duplex assay for wild boar (*W*) and domestic pig (*D*) | | | | | | | | | | | | | | | | | | | | | |

**Supplementary Table 3:** Results of preliminary selectivity tests (n = 2) for primer/probe systems targeting chromosome 5, 9 or 13. All experiments were performed with the the QuantiTect Multiplex PCR NoROX Master Mix (Qiagen, Hilden Germany) using forward primer/ reverse primer/ probe concentrations of  200/200/100 nM. DNA extracts were used at a concentration of 10 ng/µL. Primer/probe systems Chr5a – d, Chr9a - t and Chr13a – c were developed for the identification of wild boar and primer/probe systems Chr9u – bb were developed for the identification of domestic pig.

| **System** | **Chr5a** | **Chr5b** | **Chr5c** | **Chr5d** | **Chr13a** | **Chr13b** | **Chr13c** | **Chr9a** | | **Chr9b** | | **Chr9c** | | **Chr9d** | | **Chr9e** | | **Chr9f** | | **Chr9g** | **Chr9h** | | **Chr9i** | | **Chr9j** | |
| --- | --- | --- | --- | --- | --- | --- | --- | --- | --- | --- | --- | --- | --- | --- | --- | --- | --- | --- | --- | --- | --- | --- | --- | --- | --- | --- |
| **Species** | **Ct** | **Ct** | **Ct** | **Ct** | **Ct** | **Ct** | **Ct** | **Ct** | | **Ct** | | **Ct** | | **Ct** | | **Ct** | | **Ct** | | **Ct** | **Ct** | | **Ct** | | **Ct** | |
| Wild boar | 21.40 | 36.65 | 37.43 | -^a^ | 22.69 | 22.93 | 22.61 | 20.99 | | 21.32 | | 20.64 | | 20.41 | | 20.96 | | 20.45 | | 20.55 | 22.18 | | 22.09 | | 21.78 | |
| Duroc | 21.19 | 34.50 | 32.41 | 33.16 | 23.02 | 24.68 | 26.27 | 21.66 | | 23.14 | | 21.81 | | 23.91 | | 21.50 | | 21.26 | | 21.15 | 22.66 | | 23.59 | | 27.84 | |
| Iberian | 20.76 | 34.44 | 34.72 | -^a^ | 22.30 | 23.72 | 25.11 | 20.76 | | 22.32 | | 20.64 | | 22.90 | | 20.53 | | 20.26 | | 20.38 | 21.52 | | 22.02 | | 27.02 | |
| Swabian-Hall swine | 20.98 | 35.32 | 35.12 | -^a^ | 22.89 | 24.34 | 25.97 | 21.58 | | 22.56 | | 21.37 | | 23.52 | | 21.28 | | 21.16 | | 21.21 | 22.54 | | 23.11 | | 27.88 | |
| Mangalica | 21.09 | 35.47 | 36.02 | -^a^ | 22.71 | 23.71 | 25.78 | 21.36 | | 22.39 | | 21.66 | | 23.41 | | 21.15 | | 21.18 | | 21.00 | 22.18 | | 22.93 | | 27.83 | |
| Edelschwein | 22.54 | 35.35 | 36.39 | 39.40 | 23.70 | 24.77 | 26.49 | 22.44 | | 23.28 | | 22.47 | | 24.20 | | 22.34 | | 21.79 | | 22.09 | 23.35 | | 23.55 | | 28.10 | |
| Landrace | 21.77 | 34.71 | 34.53 | -^a^ | 23.68 | 24.70 | 26.74 | 22.38 | | 22.85 | | 22.23 | | 24.11 | | 21.71 | | 21.43 | | 21.63 | 23.25 | | 23.46 | | 27.35 | |
| Edelschwein x Landrace | 21.69 | 35.58 | 35.19 | -^a^ | 23.95 | 25.06 | 26.86 | 22.85 | | 23.49 | | 22.43 | | 24.38 | | 22.43 | | 22.27 | | 22.10 | 23.52 | | 24.05 | | 28.74 | |
| Landrace x Edelschwein | 22.52 | 36.59 | 34.19 | 36.86 | 23.83 | 25.71 | 26.97 | 23.10 | | 23.81 | | 22.81 | | 24.45 | | 22.69 | | 22.32 | | 22.18 | 23.60 | | 24.03 | | 28.58 | |
| Duroc x Landrace | 22.30 | 36.60 | 34.40 | -^a^ | 24.18 | 25.86 | 27.02 | 23.09 | | 23.69 | | 22.98 | | 24.56 | | 22.65 | | 22.31 | | 22.36 | 23.83 | | 24.16 | | 28.88 | |
| Pietrain x Landrace + Edelschwein | 21.90 | 35.60 | 35.98 | -^a^ | 23.74 | 25.31 | 26.82 | 22.37 | | 23.54 | | 22.52 | | 24.46 | | 22.33 | | 22.08 | | 22.19 | 23.55 | | 23.92 | | 28.65 | |
| Pietrain | 21.45 | 35.31 | 35.44 | -^a^ | 23.67 | 23.53 | 23.60 | 21.79 | | 22.69 | | 22.02 | | 24.07 | | 21.86 | | 21.47 | | 21.48 | 22.86 | | 23.20 | | 27.84 | |
| **System** | **Chr9k** | **Chr9l** | **Chr9m** | **Chr9n** | **Chr9o** | **Chr9p** | **Chr9q** | | **Chr9r** | | **Chr9s** | | **Chr9t** | |  | |  | |  |  |  |  |  |  |  |  |
| **Species** | **Ct** | **Ct** | **Ct** | **Ct** | **Ct** | **Ct** | **Ct** | | **Ct** | | **Ct** | | **Ct** | |  | |  | |  |  |  |  |  |  |  |  |
| Wild boar | 21.56 | 21.86 | 21.74 | 21.81 | 23.95 | 21.66 | 22.02 | | 22.09 | | 28.27 | | 25.82 | |  | |  | |  |  |  |  |  |  |  |  |
| Duroc | 24.05 | 22.65 | 24.01 | 23.27 | 35.90 | 31.66 | 33.55 | | 33.39 | | -^a^ | | -^a^ | |  | |  | |  |  |  |  |  |  |  |  |
| Iberian | 23.14 | 21.60 | 23.11 | 22.54 | 34.44 | 31.03 | 32.08 | | 32.61 | | -^a^ | | 37.68 | |  | |  | |  |  |  |  |  |  |  |  |
| Swabian-Hall swine | 24.05 | 22.32 | 24.24 | 23.58 | 38.25 | 31.78 | 32.70 | | 34.04 | | -^a^ | | 33.49 | |  | |  | |  |  |  |  |  |  |  |  |
| Mangalica | 23.55 | 22.49 | 23.60 | 23.35 | 37.09 | 31.10 | 32.50 | | 33.26 | | -^a^ | | -^a^ | |  | |  | |  |  |  |  |  |  |  |  |
| Edelschwein | 24.39 | 23.01 | 24.47 | 24.09 | 36.53 | 31.87 | 32.78 | | 33.42 | | -^a^ | | -^a^ | |  | |  | |  |  |  |  |  |  |  |  |
| Landrace | 24.82 | 22.83 | 24.43 | 24.12 | 39.05 | 31.53 | 32.99 | | 34.31 | | -^a^ | | -^a^ | |  | |  | |  |  |  |  |  |  |  |  |
| Edelschwein x Landrace | 25.26 | 23.41 | 24.92 | 24.64 | 38.09 | 31.82 | 33.63 | | 34.97 | | -^a^ | | -^a^ | |  | |  | |  |  |  |  |  |  |  |  |
| Landrace x Edelschwein | 25.39 | 23.53 | 24.89 | 24.83 | 36.68 | 31.58 | 33.25 | | 35.05 | | -^a^ | | 37.83 | |  | |  | |  |  |  |  |  |  |  |  |
| Duroc x Landrace | 25.58 | 23.64 | 25.00 | 24.43 | 36.54 | 32.07 | 33.20 | | 34.48 | | -^a^ | | 10.97 | |  | |  | |  |  |  |  |  |  |  |  |
| Pietrain x Landrace + Edelschwein | 24.80 | 23.54 | 25.23 | 24.13 | 39.01 | 32.79 | 33.87 | | 34.39 | | -^a^ | | -^a^ | |  | |  | |  |  |  |  |  |  |  |  |
| Pietrain | 24.30 | 22.87 | 24.61 | 23.56 | 36.33 | 32.47 | 33.09 | | 33.61 | | -^a^ | | 39.85 | |  | |  | |  |  |  |  |  |  |  |  |
| **System** | **Chr9u** | **Chr9v** | **Chr9w** | **Chr9x** | **Chr9y** | **Chr9z** | **Chr9aa** | | **Chr9ab** | | **Chr9ac** | | **Chr9ad** | | **Chr9ae** | | **Chr9af** | | **Chr9ag** | **Chr9ah** | **Chr9ai** | **Chr9aj** | | **Chr9ak** | |  |
| **Species** | **Ct** | **Ct** | **Ct** | **Ct** | **Ct** | **Ct** | **Ct** | | **Ct** | | **Ct** | | **Ct** | | **Ct** | | **Ct** | | **Ct** | **Ct** | **Ct** | **Ct** | | **Ct** | |  |
| Landrace x Edelschwein | 22.81 | 22.93 | 22.76 | 22.84 | 22.89 | 24.11 | 23.14 | | 22.90 | | 25.58 | | 24.15 | | 23.11 | | 24.39 | | 25.21 | 23.76 | 23.83 | 22.86 | | 28.75 | |  |
| Iberian | 20.35 | 20.18 | 20.19 | 20.37 | 20.16 | 21.53 | 20.98 | | 20.39 | | 23.31 | | 21.90 | | 20.83 | | 22.13 | | 22.99 | 21.14 | 21.40 | 20.40 | | 26.15 | |  |
| Wild boar | 20.70 | 21.51 | 21.09 | 21.69 | 29.38 | 30.64 | 30.81 | | 28.31 | | 30.41 | | 30.69 | | 28.26 | | 30.51 | | 32.67 | 31.68 | 29.68 | 29.71 | | 38.72 | |  |
| **System** | **Chr9al** | **Chr9am** | **Chr9an** | **Chr9ao** | **Chr9ap** | **Chr9aq** | **Chr9ar** | | **Chr9as** | | **Chr9at** | | **Chr9au** | | **Chr9av** | | **Chr9aw** | | **Chr9ax** | **Chr9ay** | **Chr9az** | **Chr9ba** | | **Chr9bb** | |  |
| **Species** | **Ct** | **Ct** | **Ct** | **Ct** | **Ct** | **Ct** | **Ct** | | **Ct** | | **Ct** | | **Ct** | | **Ct** | | **Ct** | | **Ct** | **Ct** | **Ct** | **Ct** | | **Ct** | |  |
| Landrace x Edelschwein | 28.69 | 30.50 | 36.40 | 37.08 | 39.40 | 27.05 | 31.07 | | 31.13 | | 31.46 | | 32.11 | | 31.84 | | 35.46 | | -^a^ | -^a^ | -^a^ | -^a^ | | -^a^ | |  |
| Iberian | 26.73 | 28.88 | 34.48 | 36.04 | 35.76 | 25.00 | 28.97 | | 28.93 | | 29.45 | | 30.27 | | 29.90 | | 33.89 | | 36.89 | 38.89 | -^a^ | -^a^ | | -^a^ | |  |
| Wild boar | 37.32 | -^a^ | -^a^ | -^a^ | -^a^ | 36.03 | 38.64 | | 39.95 | | -^a^ | | -^a^ | | -^a^ | | -^a^ | | -^a^ | -^a^ | -^a^ | -^a^ | | -^a^ | |  |
| ^a^ no increase in the fluorescence signal within 40 cycles | | | | | | | | | | | | | | | | | | | | | | | | | |  |

**Supplementary Table 4:** Primer and probe concentrations used in the optimisation experiments.

|  | **Combination** | **c_forward primer_ (nM)** | **c_reverse primer_ (nM)** | **c_probe_ (nM)** |  |
| --- | --- | --- | --- | --- | --- |
| **Assay_Chr9_*_W_* and assay_Chr9_*_D_*** | 1 | 50 | 50 | 25 |  |
|  | 2 | 50 | 50 | 50 |  |
|  | 3 | 100 | 100 | 50 |  |
|  | 4 | 100 | 100 | 100 |  |
|  | 5 | 100 | 100 | 250 |  |
|  | 6 | 200 | 200 | 50 |  |
|  | 7 | 200 | 200 | 100 |  |
|  | 8 | 200 | 200 | 250 |  |
|  | 9 | 350 | 350 | 50 |  |
|  | 10 | 350 | 350 | 100 |  |
|  | 11 | 350 | 350 | 250 |  |
|  | 12 | 500 | 500 | 50 |  |
|  | 13 | 500 | 500 | 100 |  |
|  | 14 | 500 | 500 | 250 |  |
|  | 15 | 700 | 700 | 50 |  |
|  | 16 | 700 | 700 | 100 |  |
|  | 17 | 700 | 700 | 250 |  |
|  | 18 | 900 | 900 | 50 |  |
|  | 19 | 900 | 900 | 100 |  |
|  | 20 | 900 | 900 | 250 |  |
|  | 21 | 12.5 | 100 | 50 |  |
|  | 22 | 25 | 100 | 50 |  |
|  | 23 | 50 | 100 | 50 |  |
|  | 24 | 3.13 | 200 | 50 |  |
|  | 25 | 6.25 | 200 | 50 |  |
|  | 26 | 12.5 | 200 | 50 |  |
|  | 27 | 25 | 200 | 50 |  |
|  | 28 | 3.13 | 300 | 50 |  |
|  | 29 | 6.25 | 300 | 50 |  |
|  | 30 | 12.5 | 300 | 50 |  |
|  | 31 | 3.13 | 400 | 50 |  |
|  | 32 | 6.25 | 400 | 50 |  |
|  | 33 | 12.5 | 400 | 50 |  |
| **Only for assay_Chr9_*_D_*** | 34 | 15.63 | 500 | 250 |  |
|  | 35 | 31.25 | 500 | 250 |  |
|  | 36 | 62.5 | 500 | 250 |  |
|  | 37 | 125 | 500 | 250 |  |
|  | 38 | 250 | 500 | 250 |  |
|  | 39 | 15.63 | 700 | 250 |  |
|  | 40 | 31.25 | 700 | 250 |  |
|  | 41 | 62.5 | 700 | 250 |  |
|  | 42 | 87.5 | 700 | 50 |  |
|  | 43 | 175 | 700 | 50 |  |
|  | 44 | 350 | 700 | 50 |  |
|  | 45 | 12.5 | 800 | 50 |  |
|  | 46 | 62.5 | 800 | 50 |  |
|  | 47 | 62.5 | 900 | 250 |  |
|  | 48 | 62.5 | 1100 | 250 |  |
| **Assay_Chr1_** | 1 | 250 | 250 | 100* |  |
|  | 2 | 250 | 250 | 200* |  |
|  | 3 | 250 | 250 | 300* |  |
|  | 4 | 500 | 500 | 100* |  |
|  | 5 | 500 | 500 | 200* |  |
|  | 6 | 500 | 500 | 300* |  |
|  | 7 | 1000 | 1000 | 100* |  |
|  | 8 | 1000 | 1000 | 200* |  |
|  | 9 | 1000 | 1000 | 300* |  |
| c… concentration; * Concentration used for the wild boar- and domestic pig-specific probe, each | | | | | |

**Supplementary Table 5:** Animal species used for cross-reactivity tests.

| **Species** | **Binomial name** | **Breed/cross-breed or country** | **Number of analysed samples** | |
| --- | --- | --- | --- | --- |
| **Animals** |  |  | **116** |  |
| Alpine ibex | *Capra ibex* |  | 1 |  |
| Cattle | *Bos taurus* |  | 1 |  |
| Chamois | *Rupicapra rupicapra* |  | 1 |  |
| Chicken | *Gallus gallus* |  | 1 |  |
| Crocodile | *Crocodylus niloticus* |  | 1 |  |
| Duck | *Anatidae* |  | 1 |  |
| Elk | *Alces alces* |  | 1 |  |
| Fallow deer | *Dama dama* |  | 1 |  |
| Goat | *Capra hircus* |  | 1 |  |
| Goose | *Anserinae* |  | 1 |  |
| Hare | *Lepus europaeus* |  | 1 |  |
| Horse | *Equus caballus* |  | 1 |  |
| Kangaroo | *Macropodidae* |  | 1 |  |
| Mouflon | *Ovis orientalis* |  | 1 |  |
| Ostrich | *Struthio camelus* |  | 1 |  |
| Rabbit | *Oryctolagus cuniculus* |  | 1 |  |
| Red deer | *Cervus elaphus* |  | 1 |  |
| Reindeer | *Rangifer tarandus* |  | 1 |  |
| Roe deer | *Capreolus capreolus* |  | 1 |  |
| Sheep | *Ovis aries* |  | 1 |  |
| Sika deer | *Cervus nippon* |  | 1 |  |
| Turkey | *Meleagris gallopavo* |  | 1 |  |
| Domestic pig | *Sus scrofa domestica* |  | 64 |  |
|  |  | Angeln Saddleback | 2 |  |
|  |  | Bentheim Black Pied | 2 |  |
|  |  | Cinta Senese | 12 |  |
|  |  | Duroc | 7 |  |
|  |  | Edelschwein | 3 |  |
|  |  | Iberian | 1 |  |
|  |  | Krškopolje | 5 |  |
|  |  | Landrace | 3 |  |
|  |  | Mangalica | 6 |  |
|  |  | Pietrain | 1 |  |
|  |  | Husum Red Pied | 2 |  |
|  |  | Saddleback | 2 |  |
|  |  | Swabian-Hall | 1 |  |
|  |  | Turopolje | 8 |  |
|  |  | Duroc x Hampshire | 2 |  |
|  |  | Duroc x Landrace | 1 |  |
|  |  | Duroc x Pietrain | 2 |  |
|  |  | Edelschwein x Landrace | 1 |  |
|  |  | Landrace x Edelschwein | 1 |  |
|  |  | Pietrain x (Landrace + Edelschwein) | 1 |  |
|  |  | Supermarket | 1 |  |
| Wild boar | *Sus scrofa scrofa* |  | 30 |  |
|  |  | Austria | 12 |  |
|  |  | Estonia | 2 |  |
|  |  | Germany | 11 |  |
|  |  | Romania | 1 |  |
|  |  | USA | 3 |  |
|  |  | Europe | 1 |  |

**Supplementary Table 6:** Plant species used for cross-reactivity tests.

| **Species** | **Binomial name** | **Number of analysed samples** |
| --- | --- | --- |
| **Plants** |  | **50** |
| Allspice | *Pimenta dioica* | 1 |
| Anise | *Pimpinella anisum* | 1 |
| Bay leaf | *Laurus nobilis* | 1 |
| Bean | *Phaseolus vulgaris* | 1 |
| Black mustard | *Brassica nigra* | 1 |
| Black pepper | *Piper nigrum* | 1 |
| Broccoli | *Brassica oleracea* | 1 |
| Buckwheat | *Fagopyrum esculentum* | 1 |
| Caraway | *Carum carvi* | 1 |
| Cardamom | *Elettaria cardamomum* | 1 |
| Carrot | *Daucus carota* | 1 |
| Celery | *Apium graveolens* | 1 |
| Chili pepper | *Capsicum sp.* | 1 |
| Chives | *Allium schoenoprasum* | 1 |
| Coriander | *Coriandrum sativum* | 1 |
| Cumin | *Cuminum cyminum* | 1 |
| Curcuma | *Curcuma longa/domestica* | 1 |
| Dill | *Anethum graveolens* | 1 |
| Fennel | *Foeniculum vulgare* | 1 |
| Garlic | *Allium sativum* | 1 |
| Ginger | *Zingiber officinale* | 1 |
| Green pea | *Pisum sativum* | 1 |
| Horseradish | *Armoracia rusticana* | 1 |
| Leek | *Allium porrum* | 1 |
| Lentil | *Lens culinaris* | 1 |
| Lovage | *Levisticum officinale* | 1 |
| Maize | *Zea mays* | 1 |
| Marjoram | *Origanum majorana* | 1 |
| Onion | *Allium cepa* | 1 |
| Oregano | *Origanum vulgare* | 1 |
| Parsley | *Petroselinum crispum* | 1 |
| Parsnip | *Pastinaca sativa* | 1 |
| Peanut | *Arachis hypogaea* | 1 |
| Pearl millet | *Pennisetum glaucum* | 1 |
| Potato | *Solanum tuberosum* | 1 |
| Radish | *Raphanus sativus* | 1 |
| Rapeseed | *Brassica napus* | 1 |
| Rice | *Oryza sativa* | 1 |
| Rosemary | *Rosmarinus officinalis* | 1 |
| Rye | *Secale cereal* | 1 |
| Sage | *Salvia officinalis* | 1 |
| Savory | *Satureja hortensis* | 1 |
| Sesame | *Sesamum indicum* | 1 |
| Sweet pepper | *Capsicum annuum* | 1 |
| Tarragon | *Artemisia dracunculus* | 1 |
| Thyme | *Thymus vulgaris* | 1 |
| Tomato | *Solanum lycopersicum* | 1 |
| Walnut | *Juglans regia* | 1 |
| Wheat | *Triticum durum* | 1 |
| White mustard | *Sinapis alba* | 1 |

**Supplementary Table 7:** Commercial master mixes, including cycling and temperature programs.

| **Reference** | **Name** | **Manufacturer** | **Cycling program** | **Temperature program** | **Addition of background dye^a^** |  |
| --- | --- | --- | --- | --- | --- | --- |
| 1 | QuantiTect Multiplex PCR NoROX Master Mix | Qiagen, | conventional | 95 °C for 15 min, 40x (94 °C 1 min, 60 °C 1 min) | + 2 µL ROX dye^c^ (25 µM) |  |
|  |  | Hilden Germany |  |  | to 1.8 mL master mix |  |
| 2 | TaqMan® Universal PCR Master Mix | Applied Biosystems, | conventional | 95 °C for 10 min, 45x (95 °C 15 s, 60 °C 1 min) | - |  |
|  |  | Foster City, CA, USA |  |  |  |  |
| 3 | GoTaq® Probe qPCR Master Mix | Promega, | fast | 95 °C for 2 min, 40x (95 °C 3 s, 60 °C 30 s)^b^ | + 2 µL CXR dye^d^ (30 µM) |  |
|  |  | Madison, Wisconsin, USA |  |  | to 1 mL master mix |  |
| 4 | PerfeCTa® qPCR ToughMix^TM^, Low ROX^TM^ | Quanta Biosciences, | fast | 95 °C for 10 min, 45x (95 °C 5 s, 60 °C 30 s) | - |  |
|  |  | Gaithersburg, Maryland, USA |  |  |  |  |
| 5 | Takyon^TM^ No Rox Probe MasterMix dTTP Blue | Eurogentec, | fast | 95 °C for 3 min, 40x (95 °C 3 s, 60 °C 30 s)^b^ | + 2 µL ROX dye (25 µM) |  |
|  |  | Seraing, Belgium |  |  | to 1 mL master mix |  |
| ^a^Addition of a background dye only necessary if the 7500 Real-time PCR cycler (Applied Biosystems) was used to master mixes that did not a priori contain a background dye. | | | | | | |
| ^b^The annealing time was increased from 30 s to 31 s if the 7500 Real-time PCR cycler (Applied Biosystems) was used. | | | | | | |
| ^c^ROX Reference dye (Invitrogen by Life Technologies, Carlsbad, CA, USA) | | | | | | |
| ^d^CXR Reference dye included in the Promega master mix kit | | | | | | |
